# Supplementary material for: Unico: a unified model for cell-type resolution genomics from heterogeneous omics data
Source: Genome Biol. 2025 Oct 3;26:333. doi: 10.1186/s13059-025-03776-3 (PMC12492706; doi:10.1186/s13059-025-03776-3)
Supplement: Supplementary file 1 — Additional file 1. Supplementary Figs. S1-S38. [file 13059_2025_3776_MOESM1_ESM.pdf]

Unico: a unified model for cell-type resolution genomics from heterogeneous omics data
Supplementary Figures

Zeyuan Johnson Chen[*,1,2], Elior Rahmani [†*,2], Eran Halperin [‡2]

[*]These authors contributed equally

[1]Department of Computer Science, University of California, Los Angeles, CA, USA

[2]Department of Computational Medicine, University of California, Los Angeles, CA, USA

[†]Corresponding Author: eliorrahmani@mednet.ucla.edu

[‡]Corresponding Author: ehalperin@cs.ucla.edu

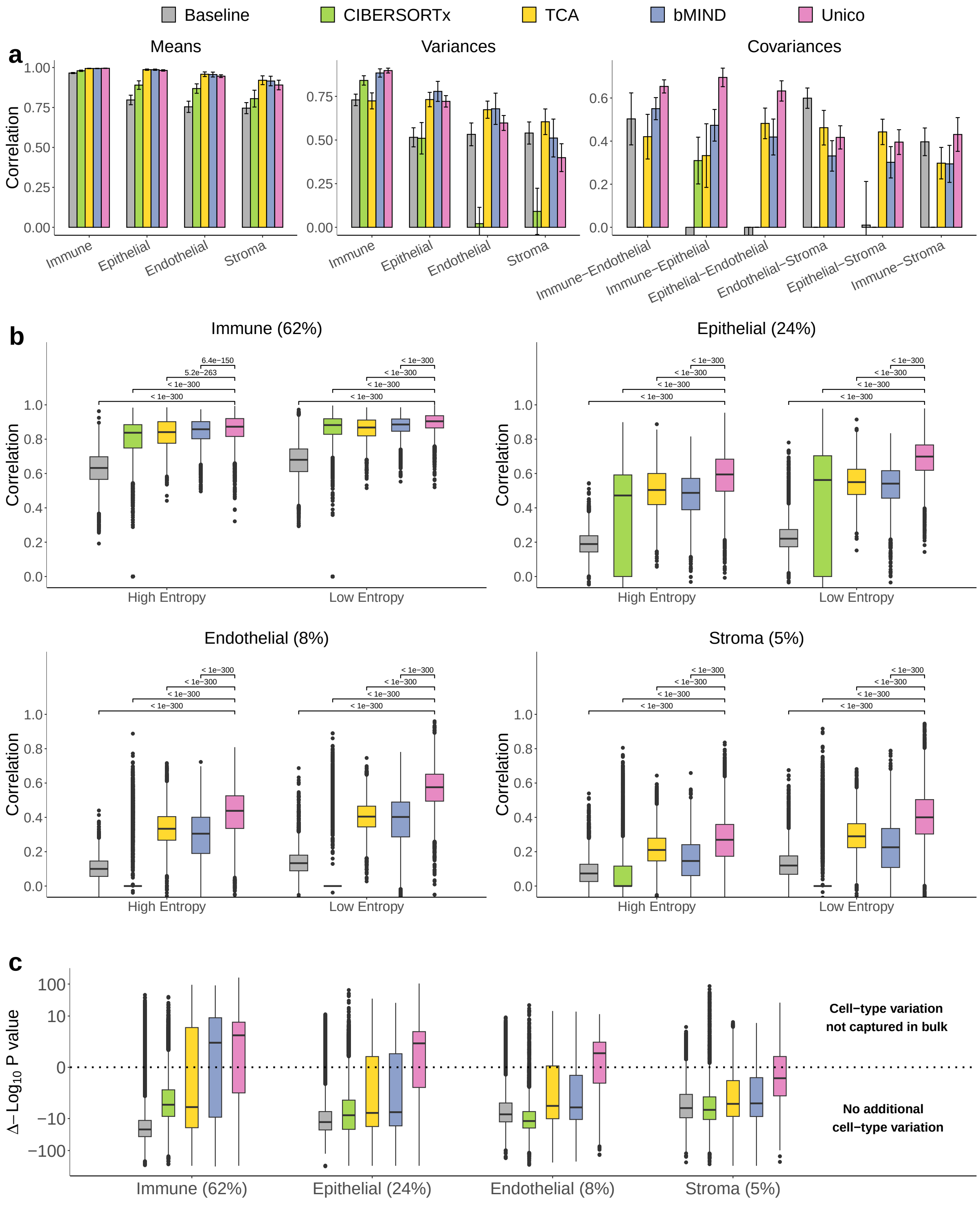

Fig. S1: Evaluation of deconvolution methods on RNA pseudo-bulk mixtures based on lung scRNA-seq dataset. (a-c) Same analyses as in main Fig. 2a-c, only using pseudo-bulk mixtures from lung scRNA-seq profiles of four cell types (500 samples and 600 genes in each set).

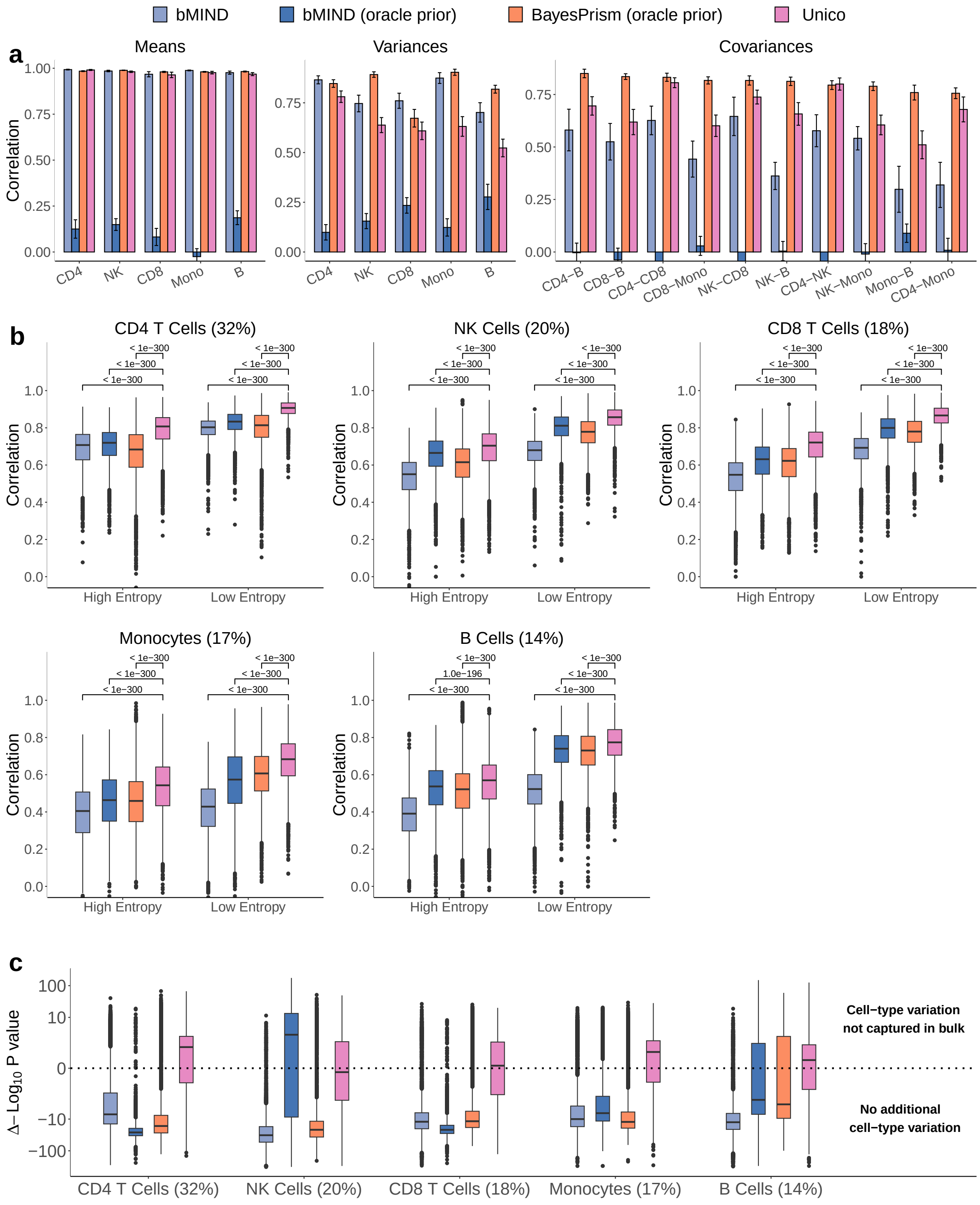

Fig. S2: Evaluation of deconvolution methods on RNA pseudo-bulk mixtures based on PBMC scRNA-seq dataset. (a-c) Same analyses as in main Fig. 2a-c, but also presenting bMIND and BayesPrism in the presence of priors learned from the true cell-type levels of all scRNA-seq samples, denoted as "bMIND (oracle prior)" in dark blue, "BayesPrism (oracle prior)" in orange, along with bMIND and Unico (500 samples and 600 genes in each set).

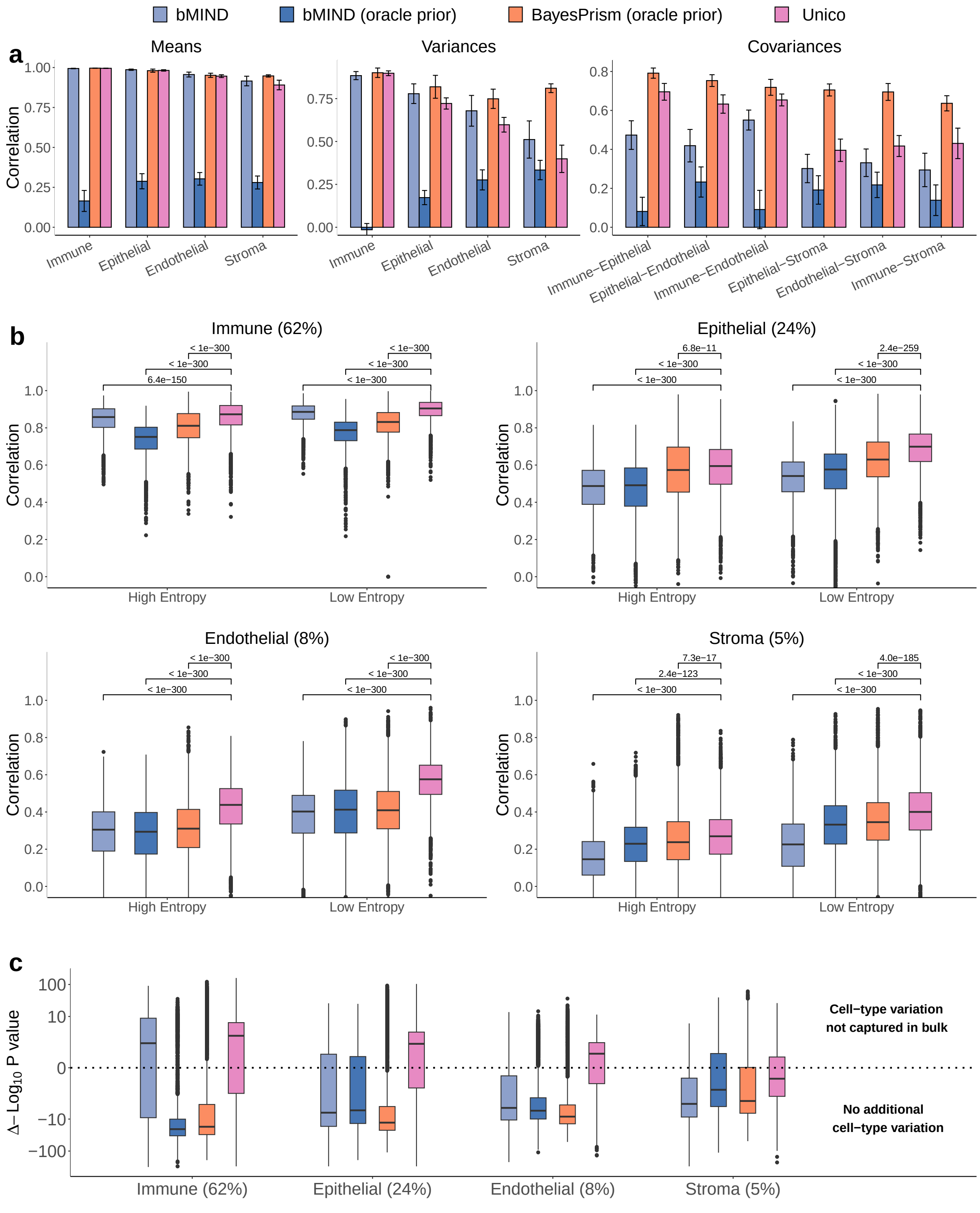

Fig. S3: Evaluation of deconvolution methods on RNA pseudo-bulk mixtures based on lung scRNA-seq dataset. (a-c) The same analyses as in Fig. S1, but also presenting bMIND and BayesPrism in the presence of priors learned from the true cell-type levels of all scRNA-seq samples, denoted as "bMIND (oracle prior)" in dark blue, "BayesPrism (oracle prior)" in orange, along with bMIND and Unico (500 samples and 600 genes in each set).

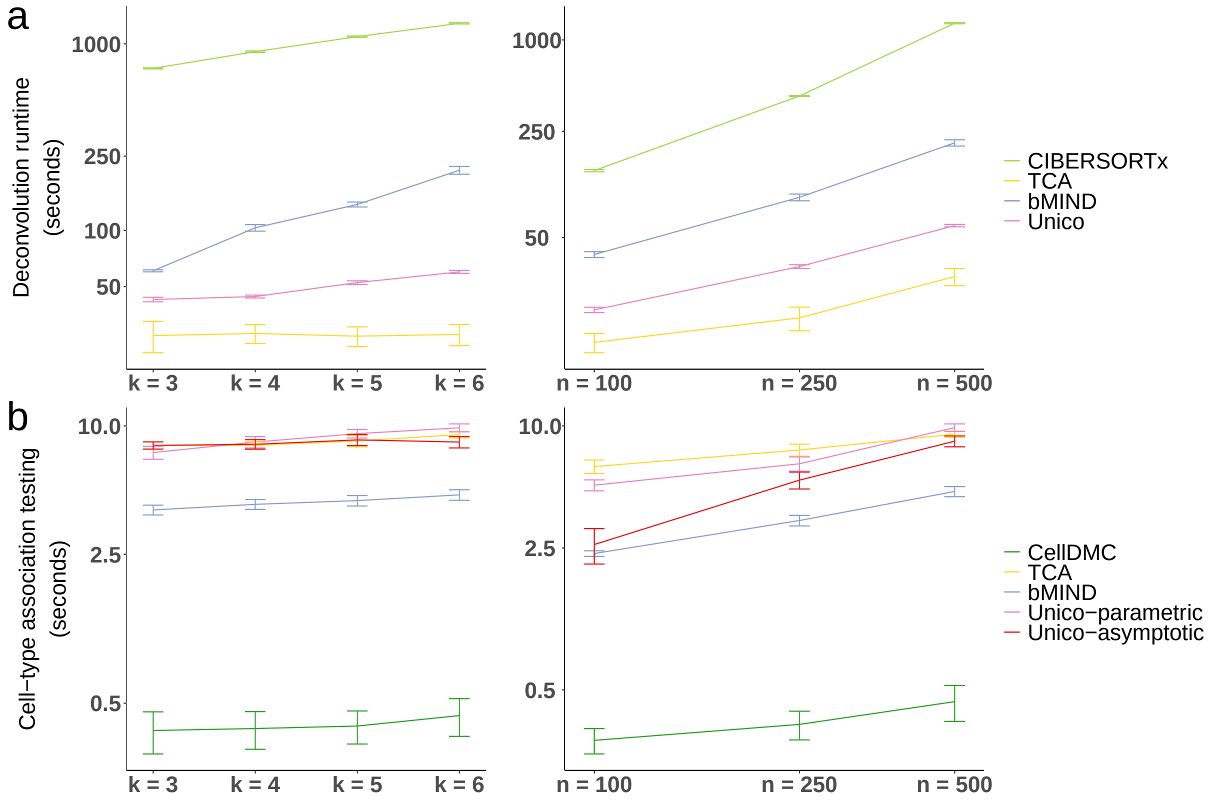

Fig. S4: Runtime of the different deconvolution methods. (a) Evaluation of deconvolution runtime on bulk methylation profiles (500 samples, 1,000 CpGs) with a varying number of cell types. (b) Evaluation of deconvolution runtime on bulk methylation profiles (six cell types, 1,000 features) with a varying number of samples. (c)-(d) Similar to (a) and (b) only for cell-type level differential methylation testing. The varied number of cell types reflects different aggregations of cell types. All methods were executed on an ARM-based Apple M1 chip with 64G RAM and in parallel on 8 computational cores. In all plots, interval bars indicate the mean and one standard deviation of the runtime across 10 simulations (log-transformed scale). Samples were drawn at random from the Hannum et al. whole-blood methylation data with $p_1 = 5$ cell-type level covariates and the top $p_2 = 10$ surrogates of technical variability as tissue-level covariates. BayesPrism was excluded from this evaluation since its runtime heavily depends on the single-cell dataset used as prior.

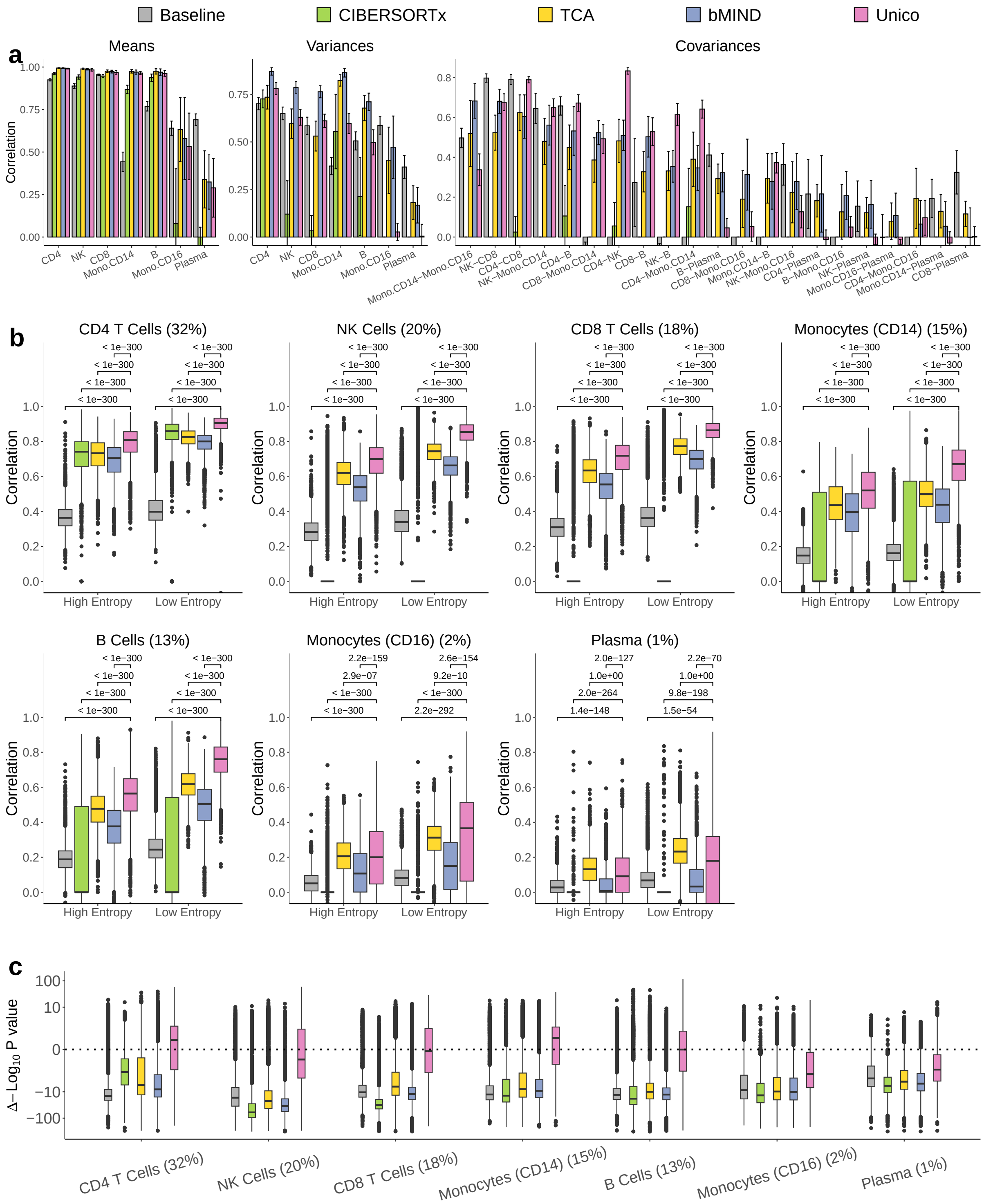

Fig. S5: Evaluation of deconvolution methods on RNA pseudo-bulk mixtures based on PBMC scRNA-seq dataset. (a-c) Same analyses as in Fig.2a-c, but using pseudo-bulk mixtures from PBMC scRNA-seq profiles of seven cell types (500 samples and 600 genes in each set).

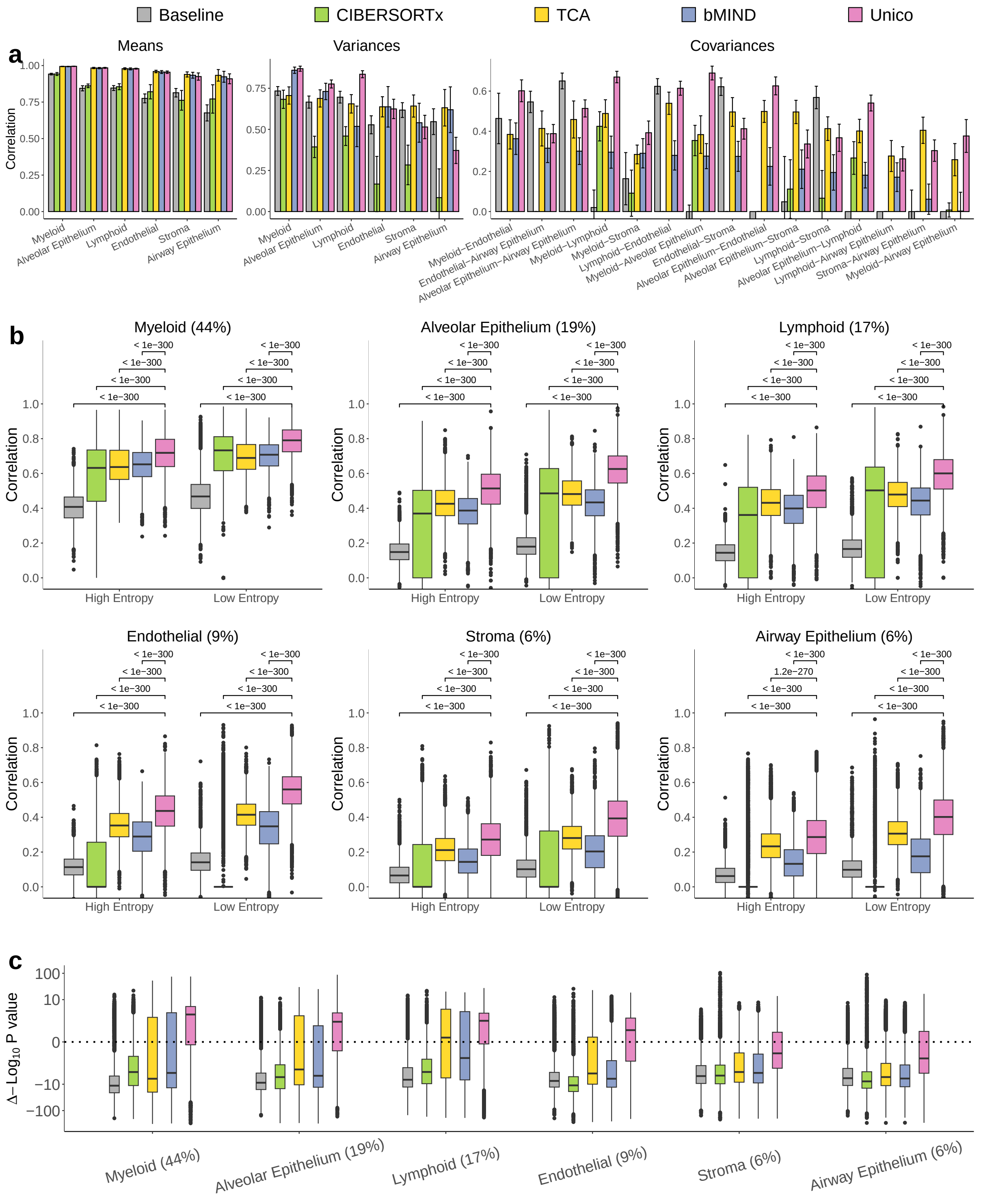

Fig. S6: Evaluation of deconvolution methods on RNA pseudo-bulk mixtures based on lung scRNA-seq dataset. (a-c) Same analyses as in Fig. S1, but using pseudo-bulk mixtures from lung scRNA-seq profiles of six cell types (500 samples and 600 genes in each set).

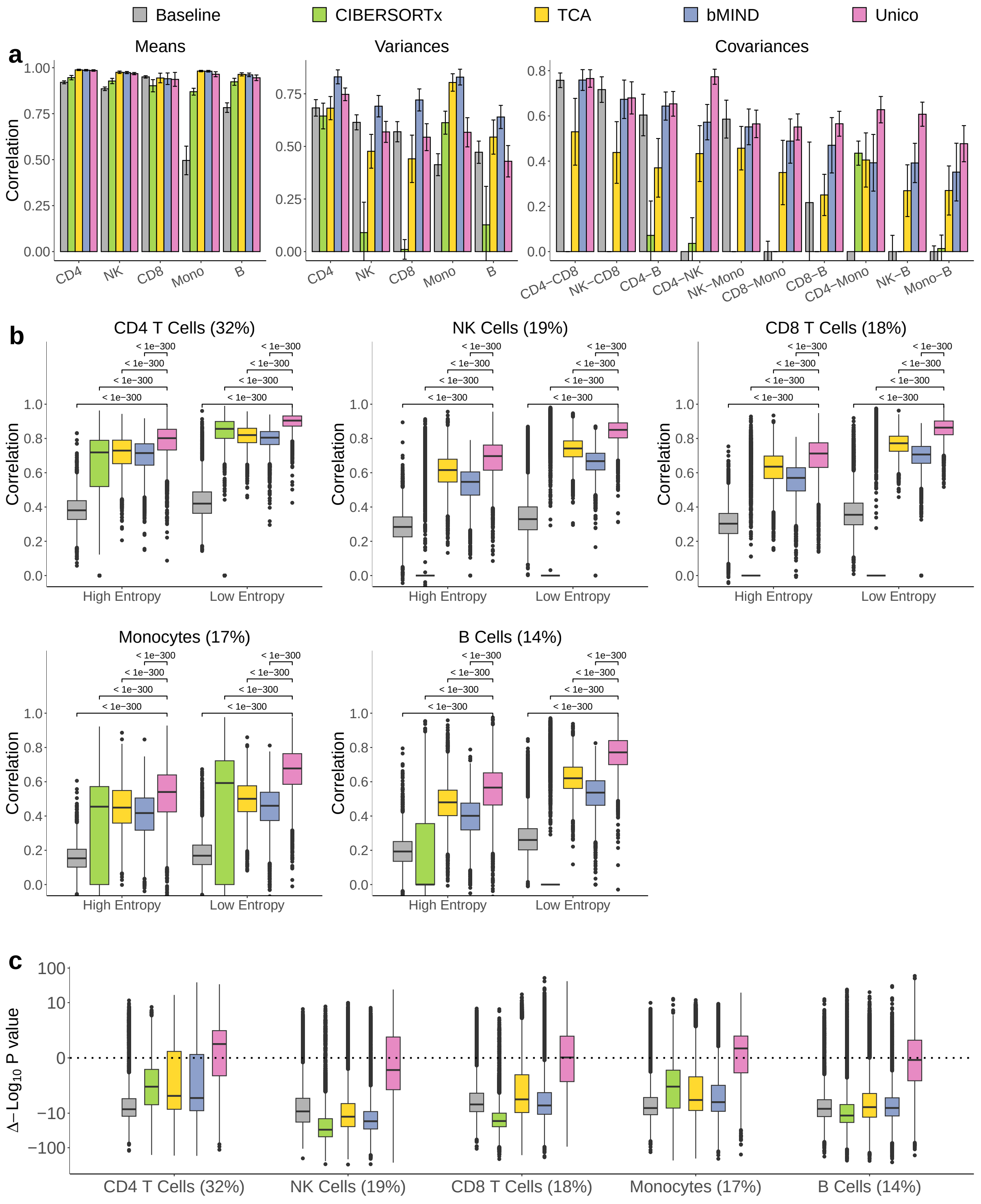

Fig. S7: Evaluation of deconvolution methods on RNA pseudo-bulk mixtures based on PBMC scRNA-seq dataset. (a-c) Same analyses as in Fig.2a-c, but using only 250 samples in pseudo-bulk mixtures from PBMC scRNA-seq profiles of five cell types (600 genes in each set).

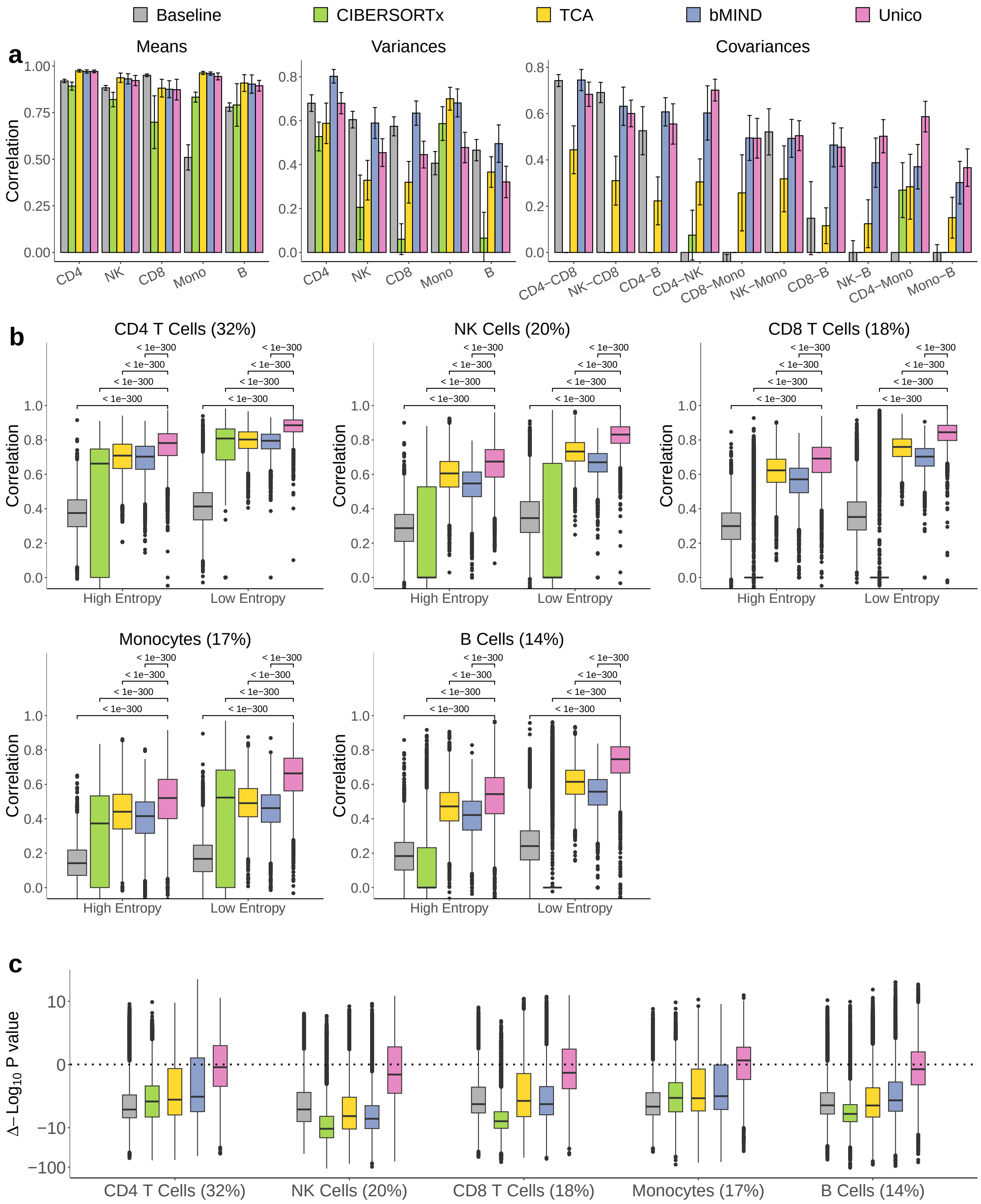

Fig. S8: Evaluation of deconvolution methods on RNA pseudo-bulk mixtures based on PBMC scRNA-seq dataset. (a-c) Same analyses as in Fig.2a-c, but using only 100 samples in pseudo-bulk mixtures from PBMC scRNA-seq profiles of five cell types (600 genes in each set).

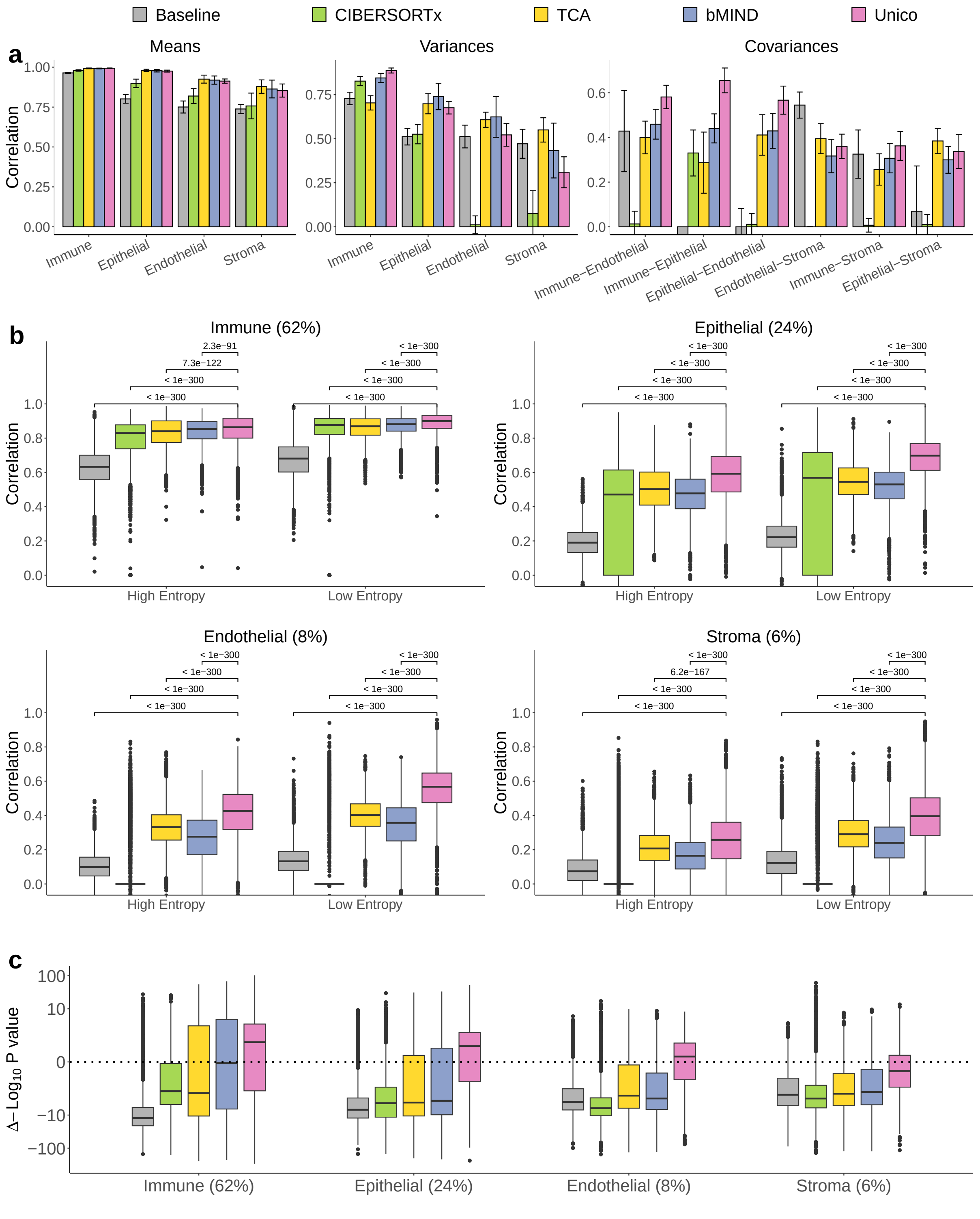

Fig. S9: Evaluation of deconvolution methods on RNA pseudo-bulk mixtures based on lung scRNA-seq dataset. (a-c) Same analyses as in Fig. S1, but using only 250 samples in pseudo-bulk mixtures from lung scRNA-seq profiles of four cell types (600 genes in each set).

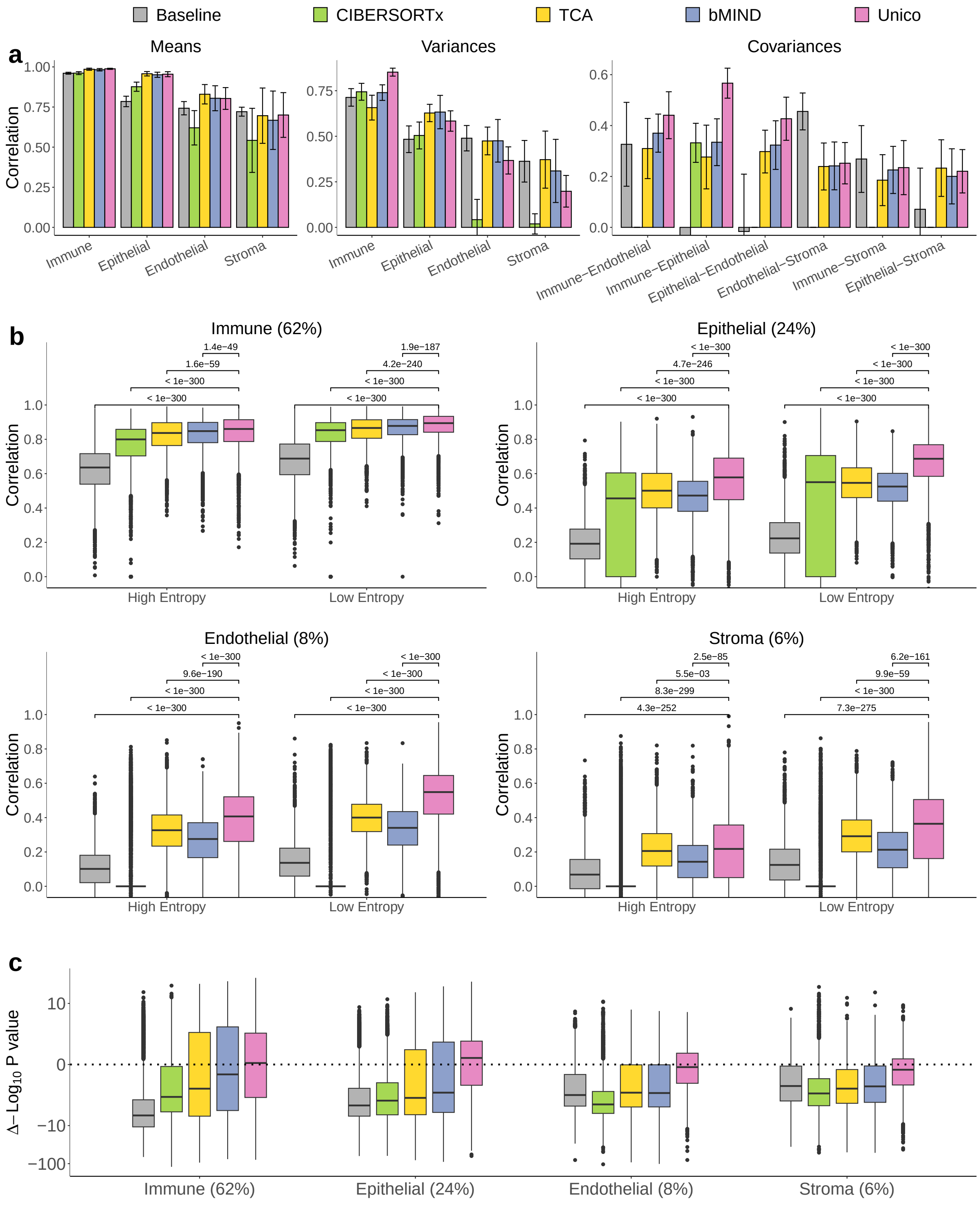

Fig. S10: Evaluation of deconvolution methods on RNA pseudo-bulk mixtures based on lung scRNA-seq dataset. (a-c) Same analyses as in Fig. S1, but using only 100 samples in pseudo-bulk mixtures from lung scRNA-seq profiles of four cell types (600 genes in each set).

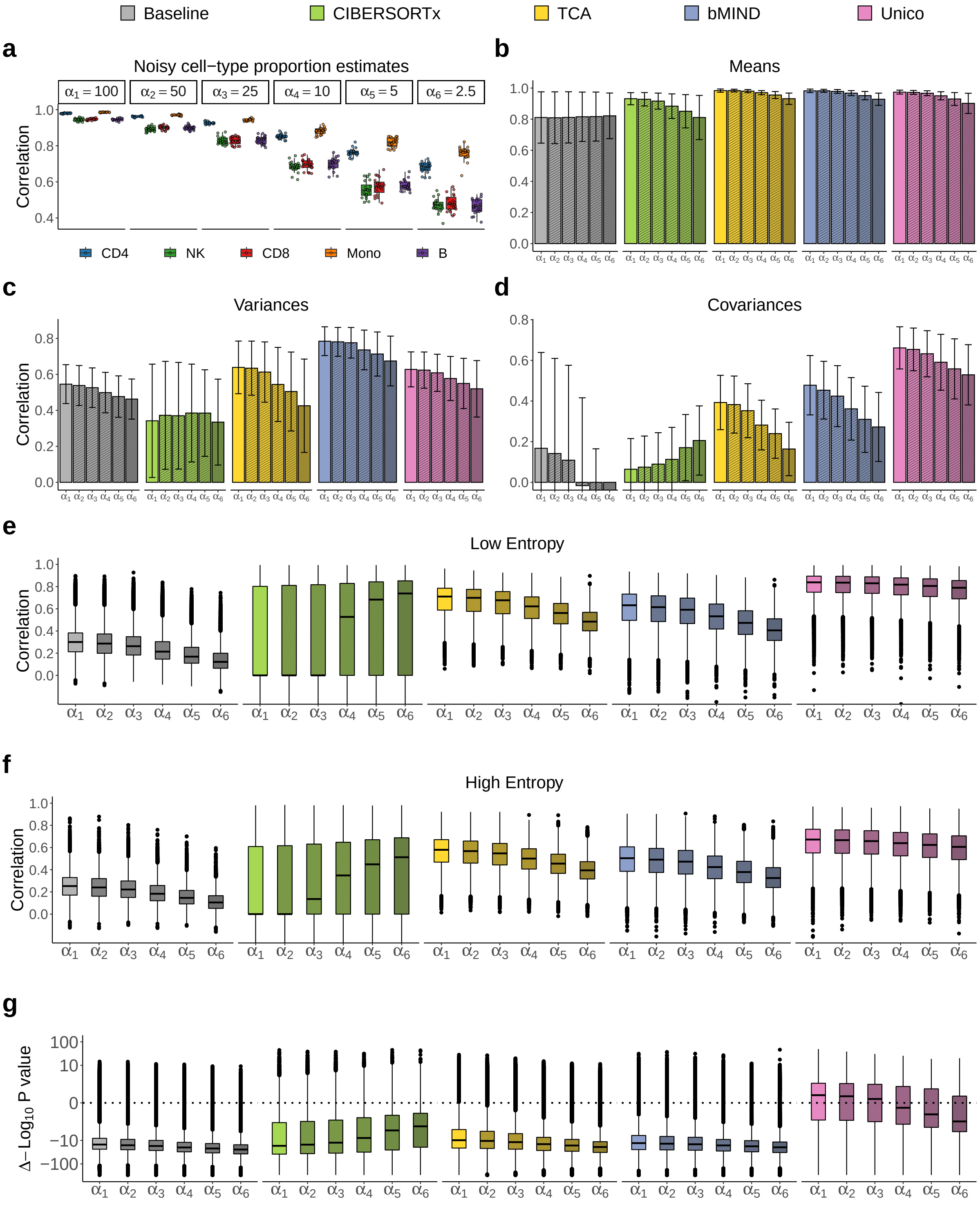

Baseline
CIBERSORTx
TCA
bMIND
Unico
a
Noisy cell−type proportion estimates
α1 = 100
α2 = 50
α3 = 25
α4 = 10
α5 = 5
α6 = 2.5
Correlation
CD4
NK
CD8
Mono
B
b
Means
c
Variances
d
Covariances
e
Low Entropy
f
High Entropy
g
Δ−Log10 P value

Fig. S11: Evaluation of deconvolution methods under varying levels of noise added to the cell-type proportions input. (a) Correlation between the ground truth cell-type proportions and the noisy version provided as input to all methods (lower α indicates more noise; Additional File 2: Supplementary Notes). (b-d) Correlation between single-cell-based estimates of population-level cell-type moments and those based on deconvolution estimates across 20 sets of pseudo-bulk mixtures from PBMC scRNA-seq profiles (500 samples and 600 genes in each set). (e-f) Evaluation of the concordance between the known cell-type profiles and the deconvolution estimates. Boxplots reflect the distribution of linear correlation across all five cell types and all genes in the low entropy (e) and high entropy (f) set across the same 20 simulations in (b-d). (g) Assessing deconvolution methods for their information that cannot be explained by pseudo bulk expression. Boxplots reflect the distribution across all cell types and genes from the same data in (b-f) of $\Delta \log_{10}$(p-value), the difference between the log-scaled p-values of the effects of the pseudo bulk expression and those of the deconvolution estimates (higher is better; Methods). All barplots and error bars in the figure represent means and one standard deviation errors; negative correlations were truncated for visualization purposes. BayesPrism is excluded as it jointly computes the cell-type fractions and the underlying 3D tensor, thus not accepting user-provided fraction estimates.

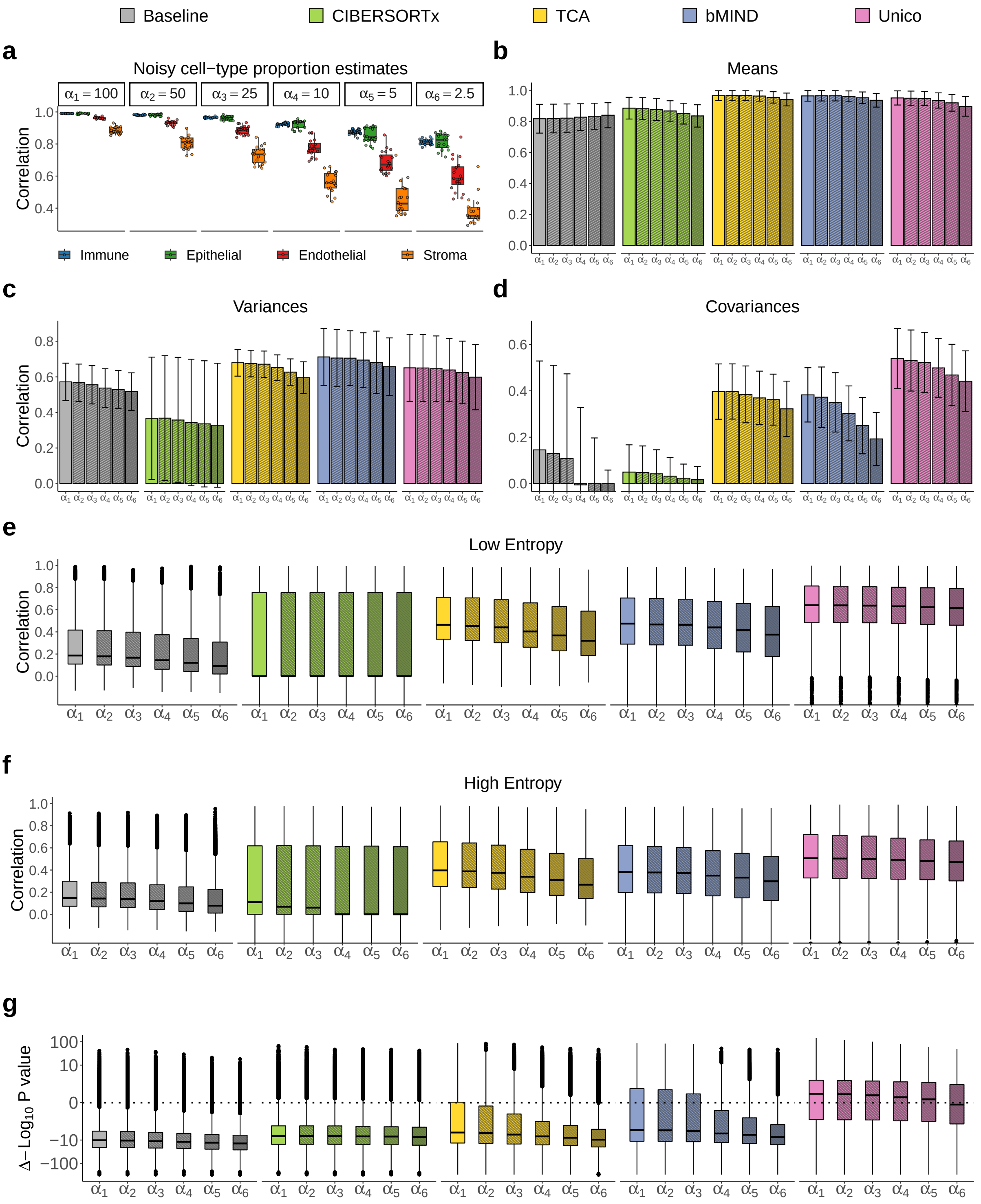

Fig. S12: Evaluation of deconvolution methods under varying levels of noise added to the cell-type proportions input. Same analyses as in Fig. S11, but on pseudo-bulk mixtures from lung scRNA-seq profiles of four cell types.

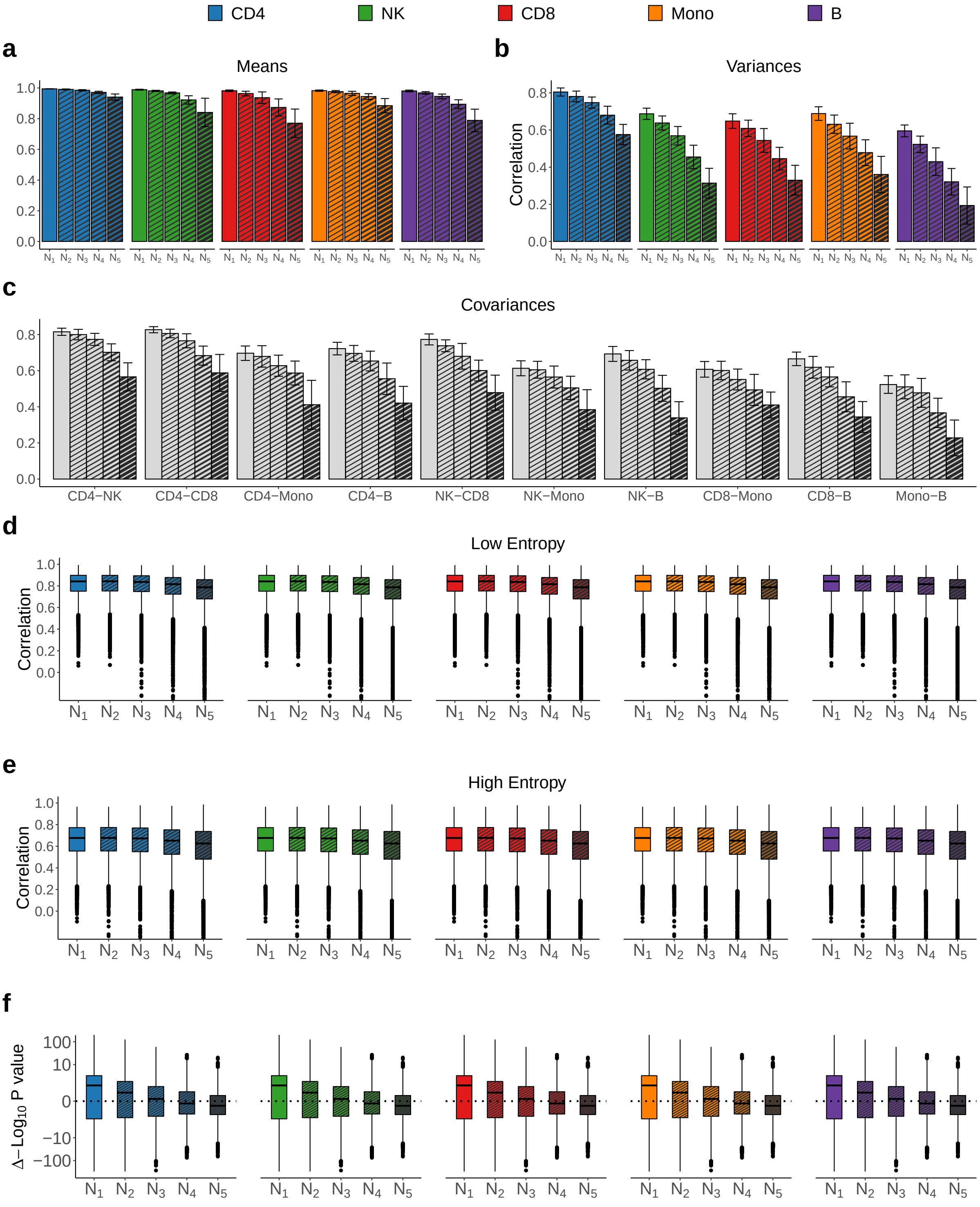
CD4
NK
CD8
Mono
B
a
Means
b
Variances
Correlation
c
Covariances
CD4−NK
CD4−CD8
CD4−Mono
CD4−B
NK−CD8
NK−Mono
NK−B
CD8−Mono
CD8−B
Mono−B
d
Low Entropy
Correlation
e
High Entropy
Correlation
f
Δ−Log10 P value
N1 N2 N3 N4 N5

Fig. S13: Evaluation of Unico's performance under varying sample sizes: N_1 = 1000, N_2 = 500, N_3 = 250, N_4 = 100, and N_5 = 50. (a-c) For each sample size, correlation between single-cell-based estimates of population-level cell-type means, variances, and covariances and those based on Unico across 20 sets of pseudo-bulk mixtures from PBMC scRNA-seq profiles (600 genes in each set). (d-e) Evaluation of the concordance between the known cell-type profiles and Unico's deconvolution estimates. Boxplots reflect the distribution of linear correlation across all five cell types and all genes in the low entropy (d) and high entropy set (e) across the same simulations in (a-c).(f) Assessing the Unico deconvolution estimates for information that cannot be explained by pseudo bulk expression. Boxplots reflect the distribution across all cell types and genes from the same data in (a-e) of $\Delta \log_{10}$(p-value), the difference between the log-scaled p-values of the effects of the pseudo bulk expression and those of the deconvolution estimates (higher is better; Methods). All barplots and error bars in the figure represent means and one standard deviation errors; negative correlations were truncated for visualization purposes.

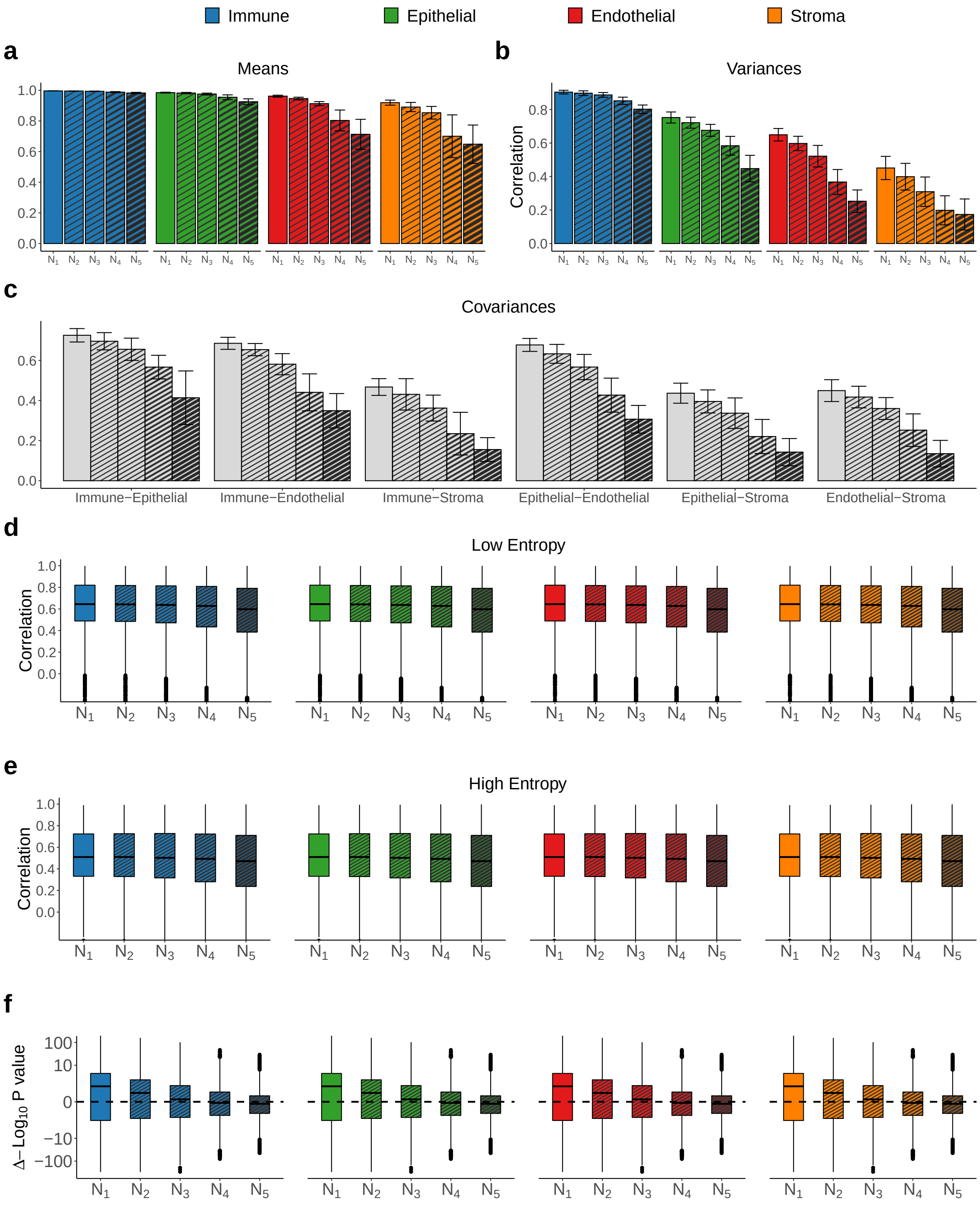

Fig. S14: Evaluation of Unico's performance under varying sample sizes: N_1 = 1000, N_2 = 500, N_3 = 250, N_4 = 100, and N_5 = 50. Same analyses as in Fig. S13, but on pseudo-bulk mixtures from lung scRNA-seq profiles of four cell types.

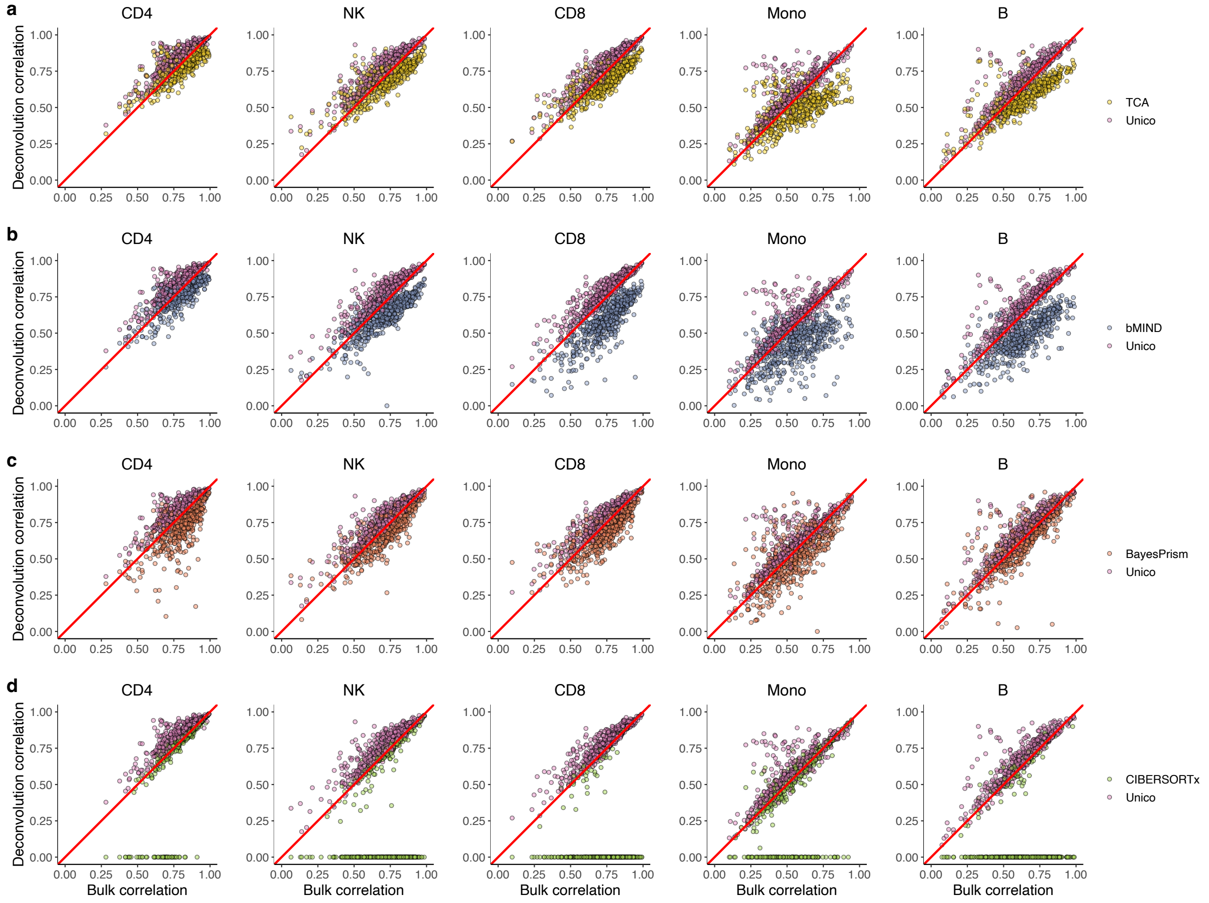

Fig. S15: Evaluation of deconvolution methods with bulk expression. Scatter plots represent the correlation between each gene's ground truth cell-type profile and its deconvolution estimates (y-axis), relative to the correlation between the ground truth profile and a bulk expression baseline (x-axis), using the same evaluation framework and pseudo-bulk mixtures from PBMC scRNA-seq profiles as in main Fig.2. Each row corresponds to pairwise comparisons between different methods: (a) TCA, (b) bMIND, (c) BayesPrism, and (d) CIBERSORTx, against Unico. Dots falling below the diagonal line indicate worse performance than the baseline.

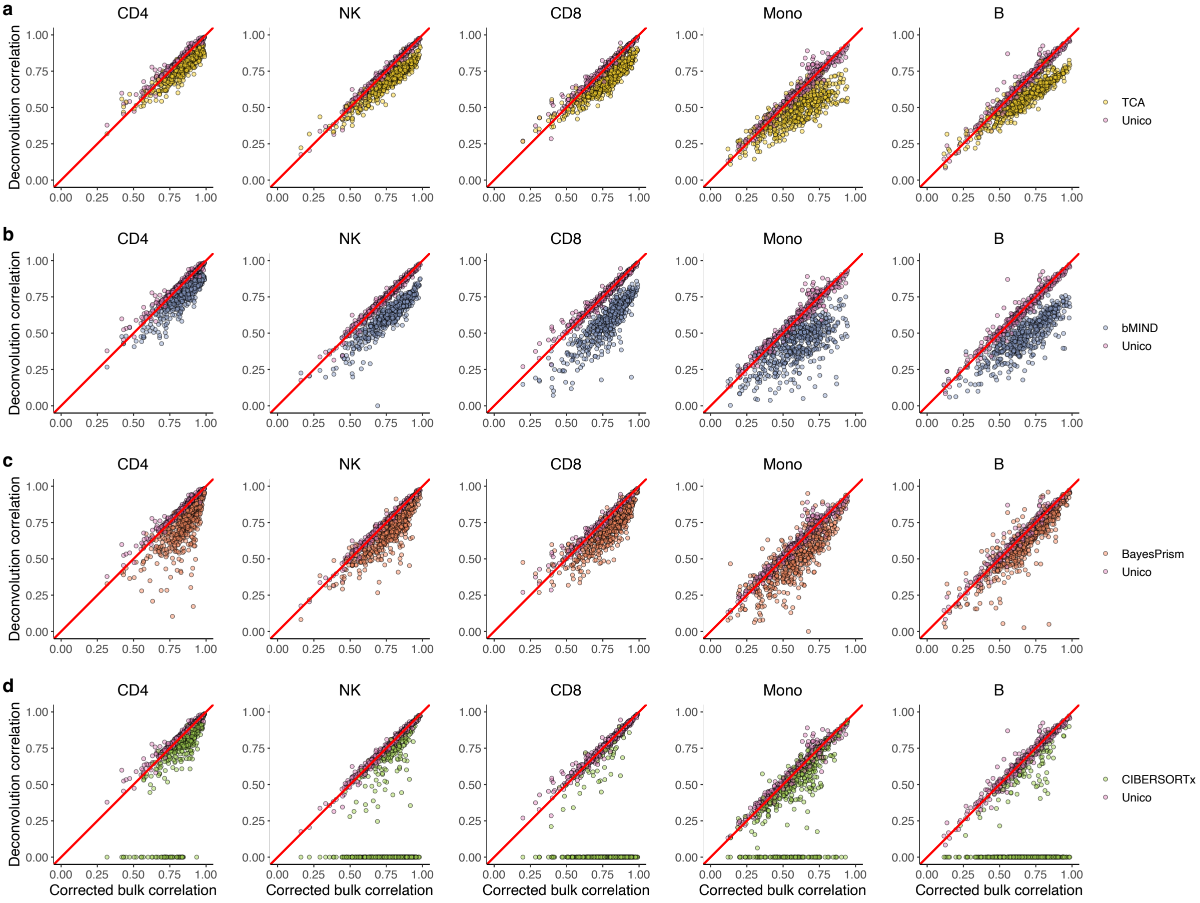

Fig. S16: Evaluation of deconvolution methods with corrected bulk expression. Scatter plots represent the correlation between each gene's ground truth cell-type profile and its deconvolution estimates (y-axis), relative to the correlation between the ground truth profile and a corrected bulk expression baseline (i.e., bulk levels with cell-type proportions regressed out; x-axis), using the same evaluation framework and pseudo-bulk mixtures from PBMC scRNA-seq profiles as in main Fig.2. Each row corresponds to pairwise comparisons between different methods: (a) TCA, (b) bMIND, (c) BayesPrism, and (d) CIBERSORTx, against Unico. Dots falling below the diagonal line indicate worse performance than the baseline.

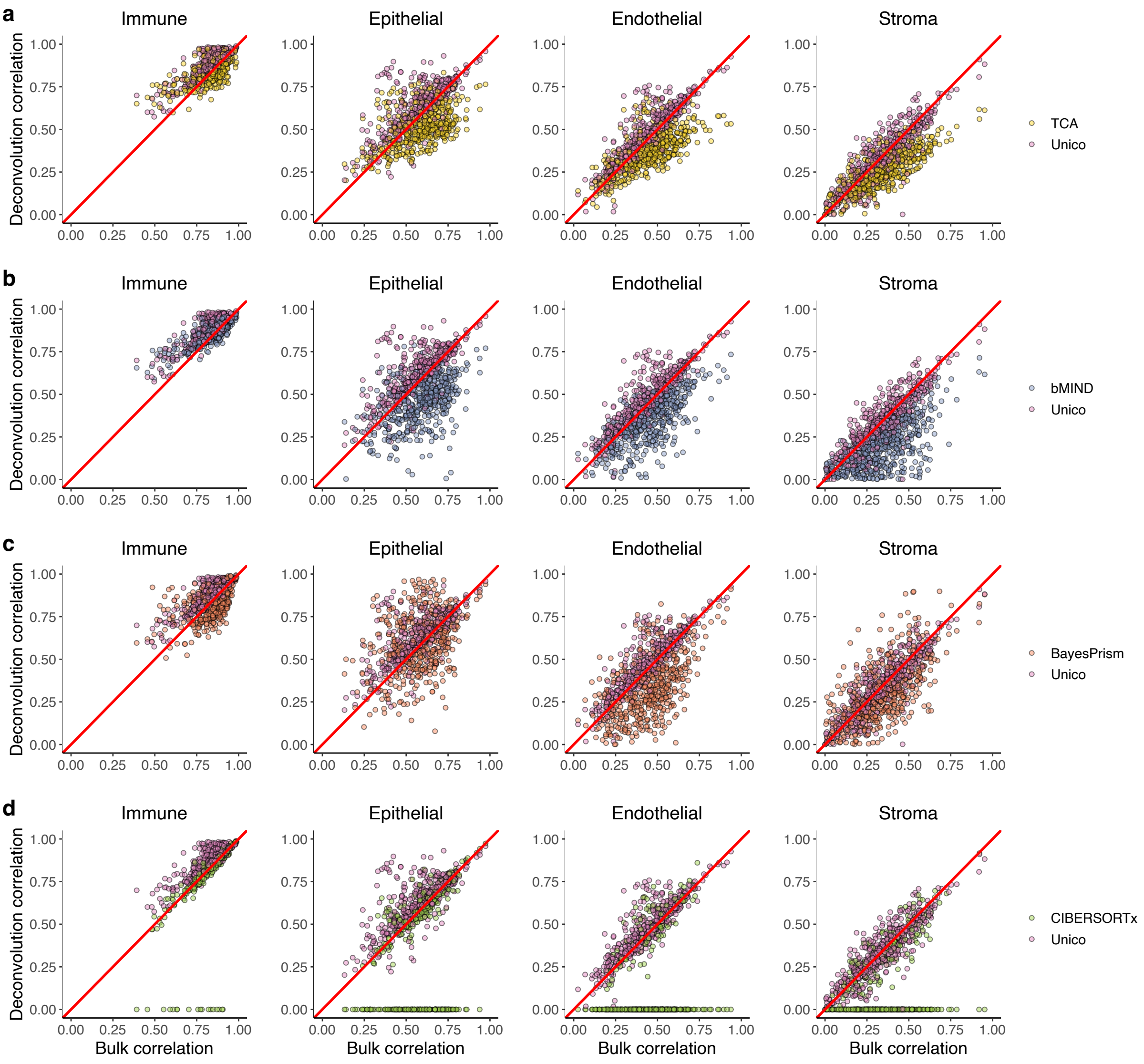

Fig. S17: Evaluation of deconvolution methods with bulk expression. Scatter plots represent the correlation between each gene's ground truth cell-type profile and its deconvolution estimates (y-axis), relative to the correlation between the ground truth profile and a bulk expression baseline (x-axis), using the same evaluation framework and pseudo-bulk mixtures from lung scRNA-seq profiles as in Fig. S1. Each row corresponds to pairwise comparisons between different methods: (a) TCA, (b) bMIND, (c) BayesPrism, and (d) CIBERSORTx, against Unico. Dots falling below the diagonal line indicate worse performance than the baseline.

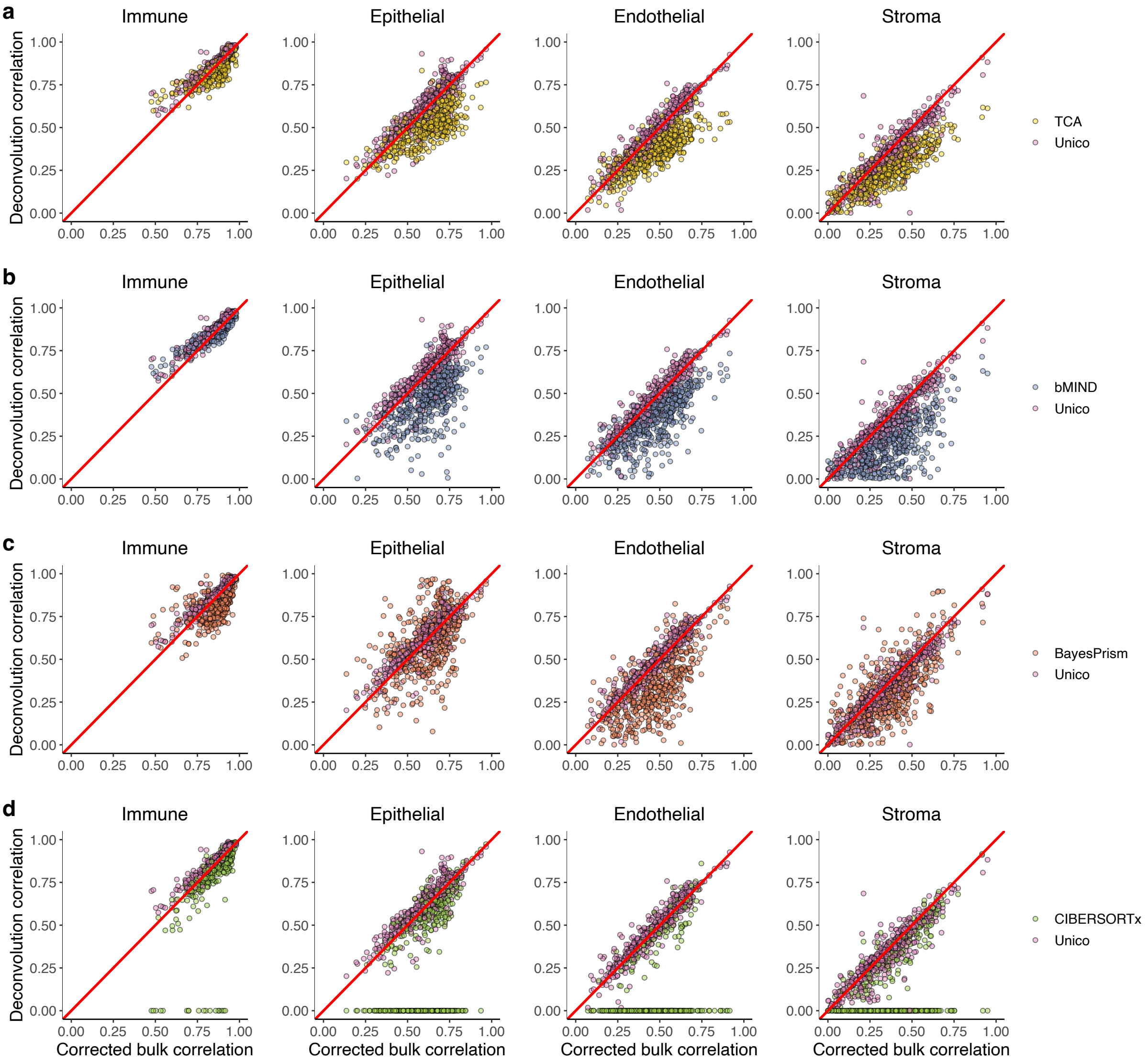

Fig. S18: Evaluation of deconvolution methods with corrected bulk expression. Scatter plots represent the correlation between each gene's ground truth cell-type profile and its deconvolution estimates (y-axis), relative to the correlation between the ground truth profile and a corrected bulk expression baseline (i.e., bulk levels with cell-type proportions regressed out; x-axis), using the same evaluation framework and pseudo-bulk mixtures from lung scRNA-seq profiles as in Fig. S1. Each row corresponds to pair-wise comparisons between different methods: (a) TCA, (b) bMIND, (c) BayesPrism, and (d) CIBERSORTx, against Unico. Dots falling below the diagonal line indicate worse performance than the baseline.

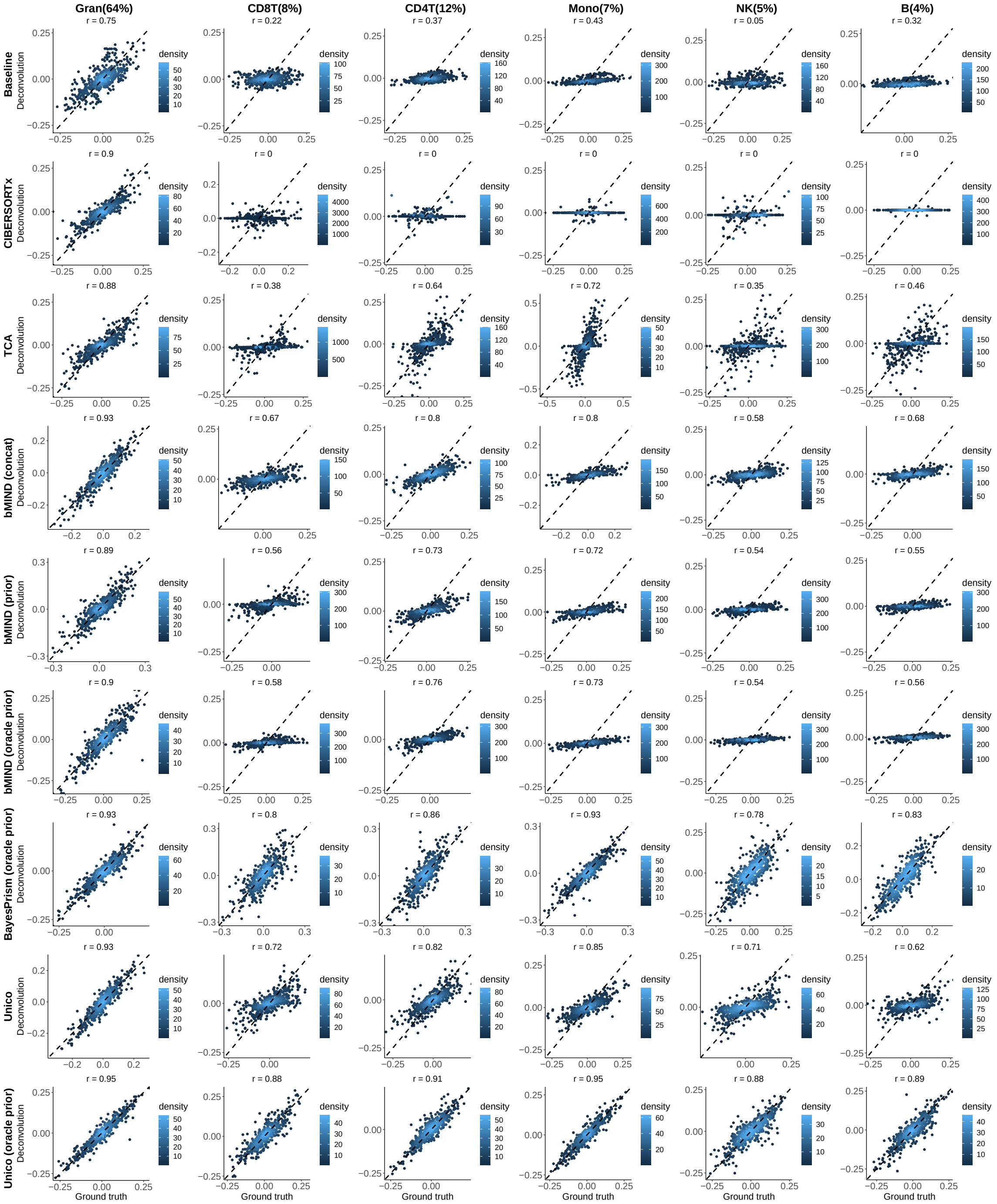

Fig. S19: Deconvolution of the low-entropy CpGs in the set of 10,000 most variable CpGs in the Reinius whole-blood DNA methylation data. Presented are experimentally measured cell-type level methylation for the whole-blood samples (values pooled across samples and CpGs per cell type; "Ground truth") and the deconvolution estimates, with different rows corresponding to different methods.

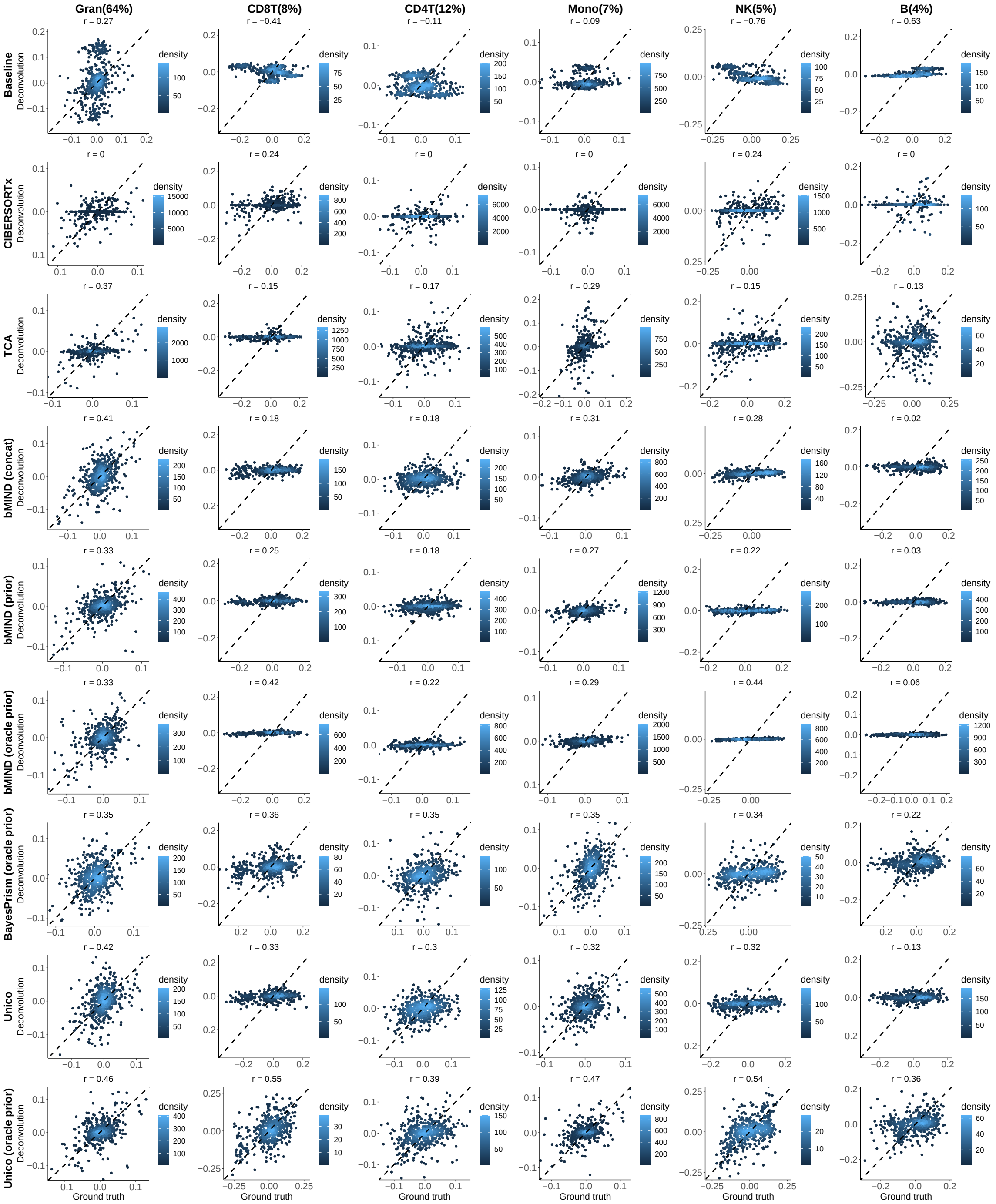

Fig. S20: Deconvolution of the high-entropy CpGs in the set of 10,000 most variable CpGs in the Reinius whole-blood DNA methylation data. Presented are experimentally measured cell-type level methylation for the whole-blood samples (values pooled across samples and CpGs per cell type; "Ground truth") and the deconvolution estimates, with different rows corresponding to different methods.

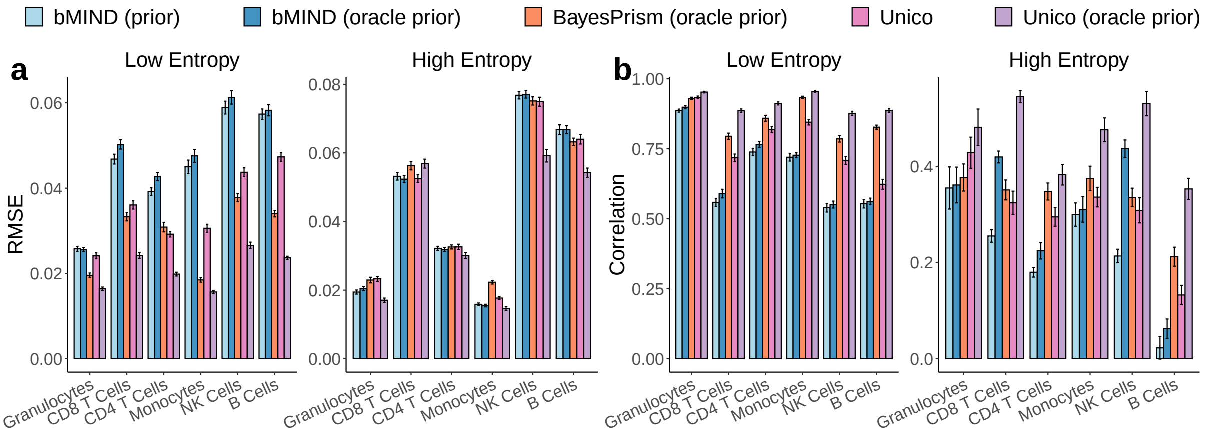

Fig. S21: Evaluation of deconvolution methods (with prior) in the set of 10,000 highly variable CpGs in the Reinius whole-blood DNA methylation data. Same analyses as in Fig. 2d and e, but on methods that can incorporate prior information. "bMIND (prior)" corresponds to using the estimated parameters from deconvolving the Hannum et al. DNA methylation dataset as prior. "(oracle prior)" indicates providing the means and covariances derived from the ground truth cell-type level profiles for bMIND and Unico, and the entire cell-type level profiles as prior for BayesPrism.

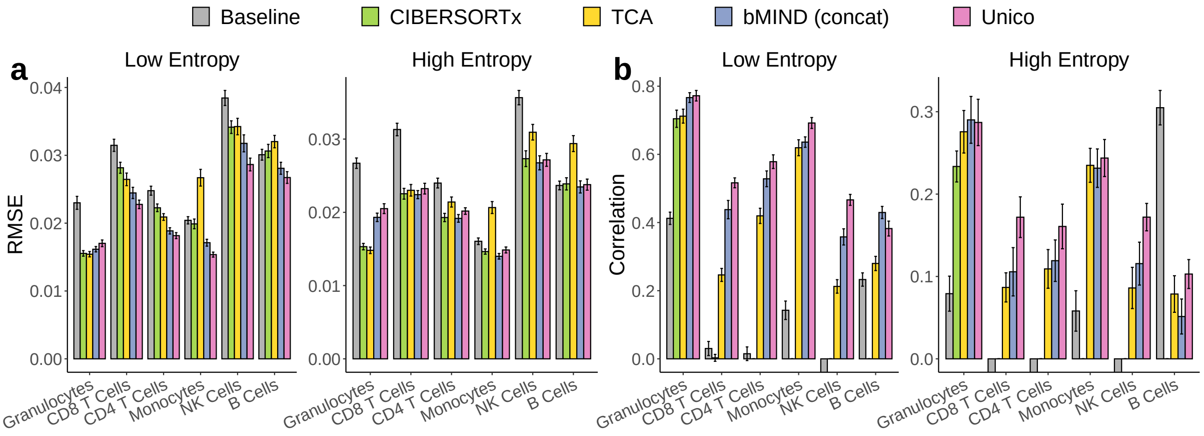

Fig. S22: Evaluation of deconvolution methods in a set of 10,000 randomly selected CpGs in the Reinius whole-blood DNA methylation data. (a-b) Evaluation in terms of RMSE (a) and correlation (b) between estimates and experimentally validated cell-type level methylation across 20 random sets of 1,000 randomly selected CpGs. Barplots and error bars in the figure represent means and one standard deviation errors; negative correlations were truncated for visualization purposes.

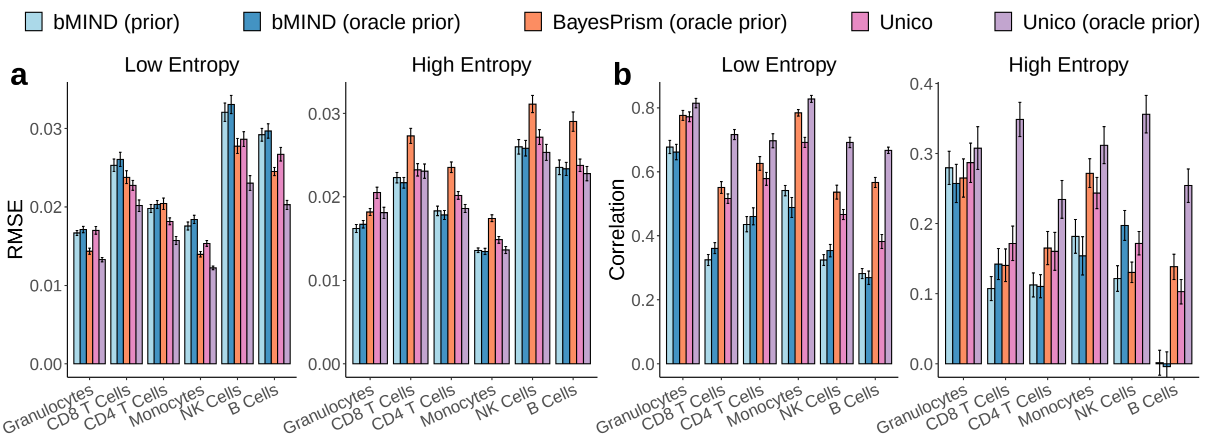

Fig. S23: Evaluation of deconvolution methods (with prior) in a set of 10,000 randomly selected CpGs in the Reinius whole-blood DNA methylation data. Same analyses as in Fig. S22, but on methods that can incorporate prior information. "bMIND (prior)" corresponds to using the estimated parameters from deconvolving the Hannum et al. DNA methylation dataset as prior. "(oracle prior)" indicates providing the means and covariances derived from the ground truth cell-type level profiles for bMIND and Unico, and the entire cell-type level profiles as prior for BayesPrism.

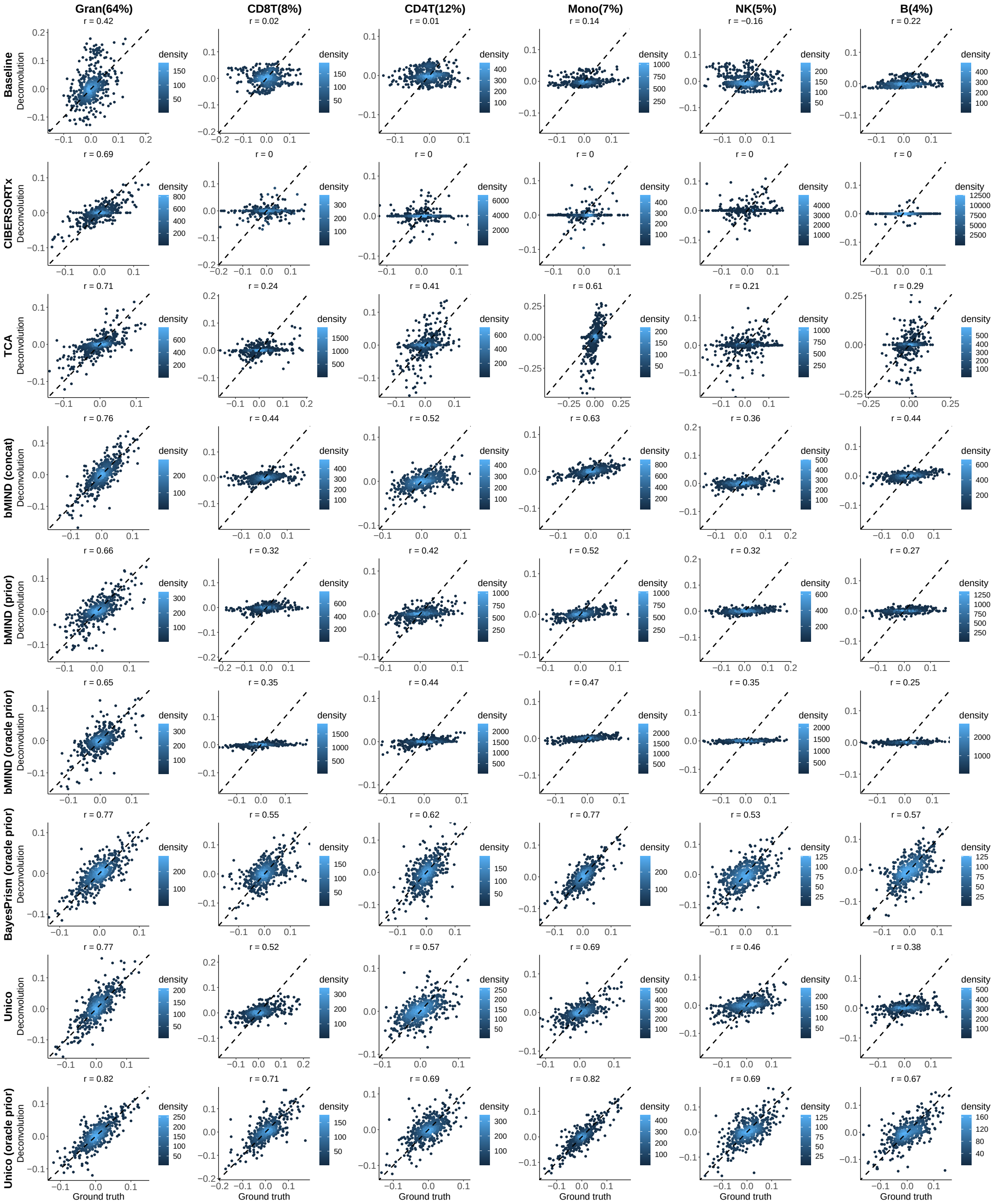

Fig. S24: Deconvolution of the low-entropy CpGs in a set of 10,000 randomly selected CpGs in the Reinius whole-blood DNA methylation data. Presented are experimentally measured cell-type level methylation for the whole-blood samples (values pooled across samples and CpGs per cell type; "Ground truth") and the deconvolution estimates, with different rows corresponding to different methods.

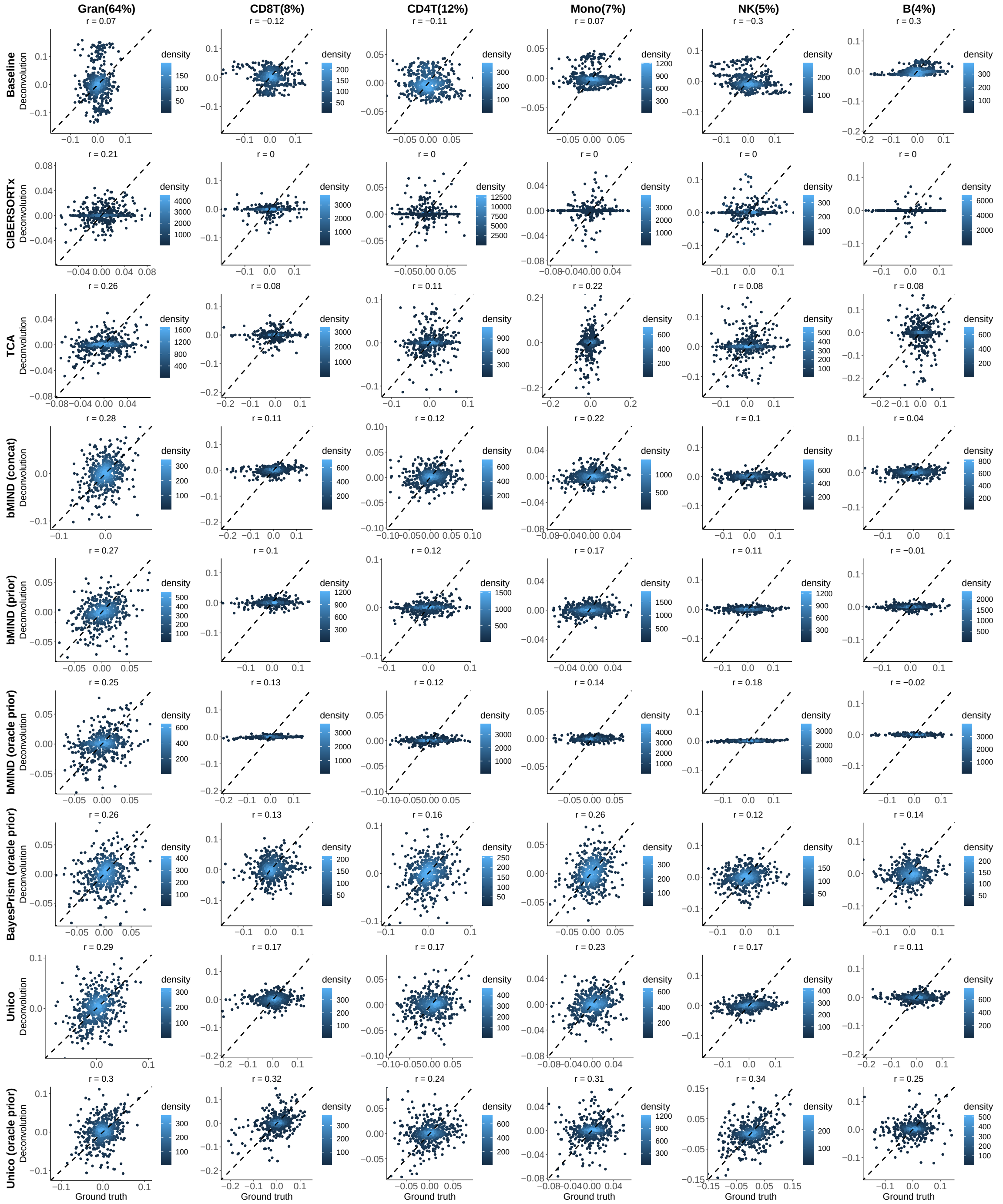

Fig. S25: Deconvolution of the high-entropy CpGs in a set of 10,000 randomly selected CpGs in the Reinius whole-blood DNA methylation data. Presented are experimentally measured cell-type level methylation for the whole-blood samples (values pooled across samples and CpGs per cell type; "Ground truth") and the deconvolution estimates, with different rows corresponding to different methods.

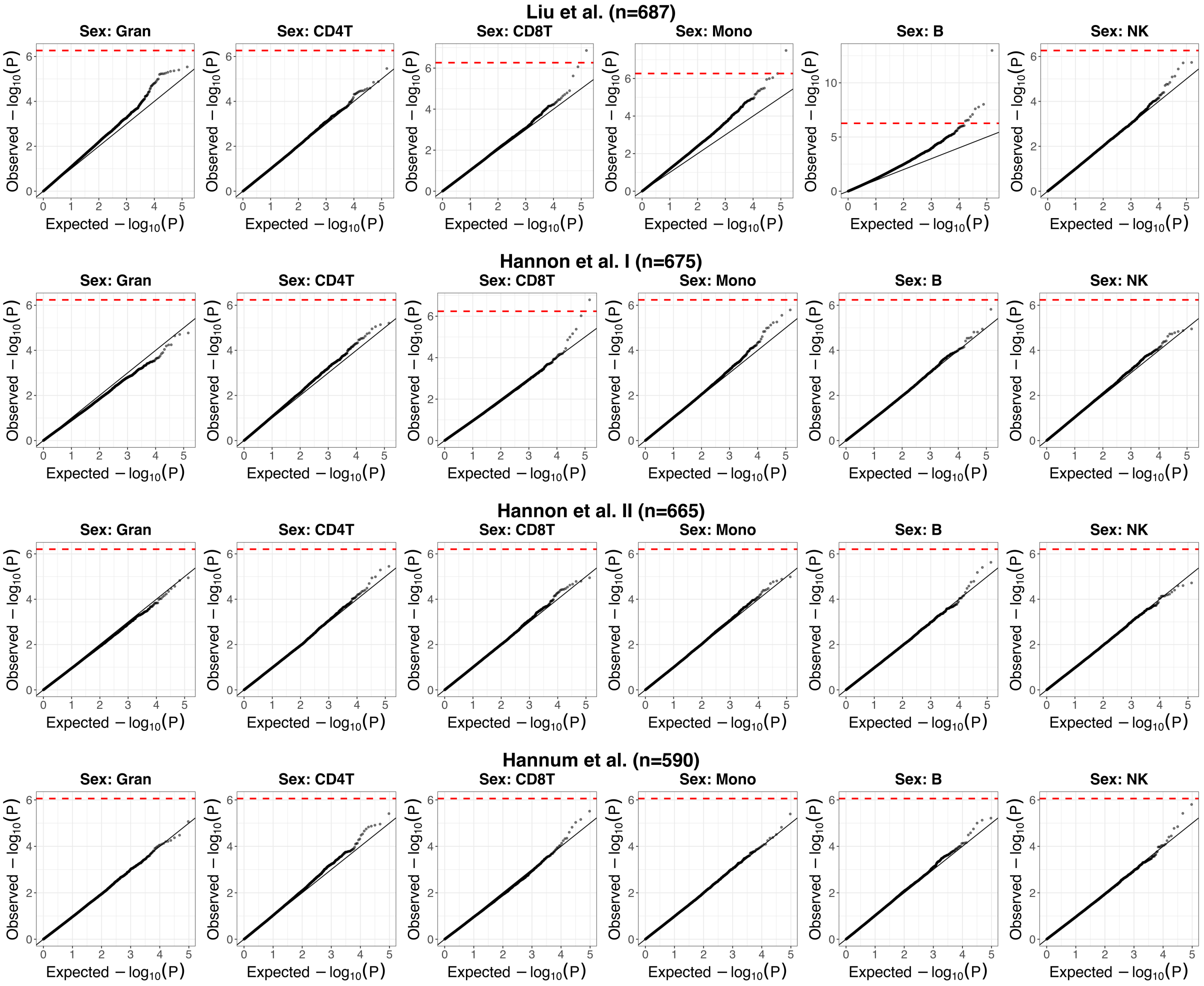

Fig. S26: Evaluation of the null distribution of Unico's asymptotically derived p-values under non-parametric testing for cell-type level differential methylation with sex in four whole-blood datasets. Presented are quantile-quantile plots with log-transformed expected p-values versus the observed p-values under permutations of the condition (i.e., under the null). Red horizontal dashed lines indicate the Bonferroni-corrected threshold, adjusting for the number of CpGs and the number of cell-types under test.

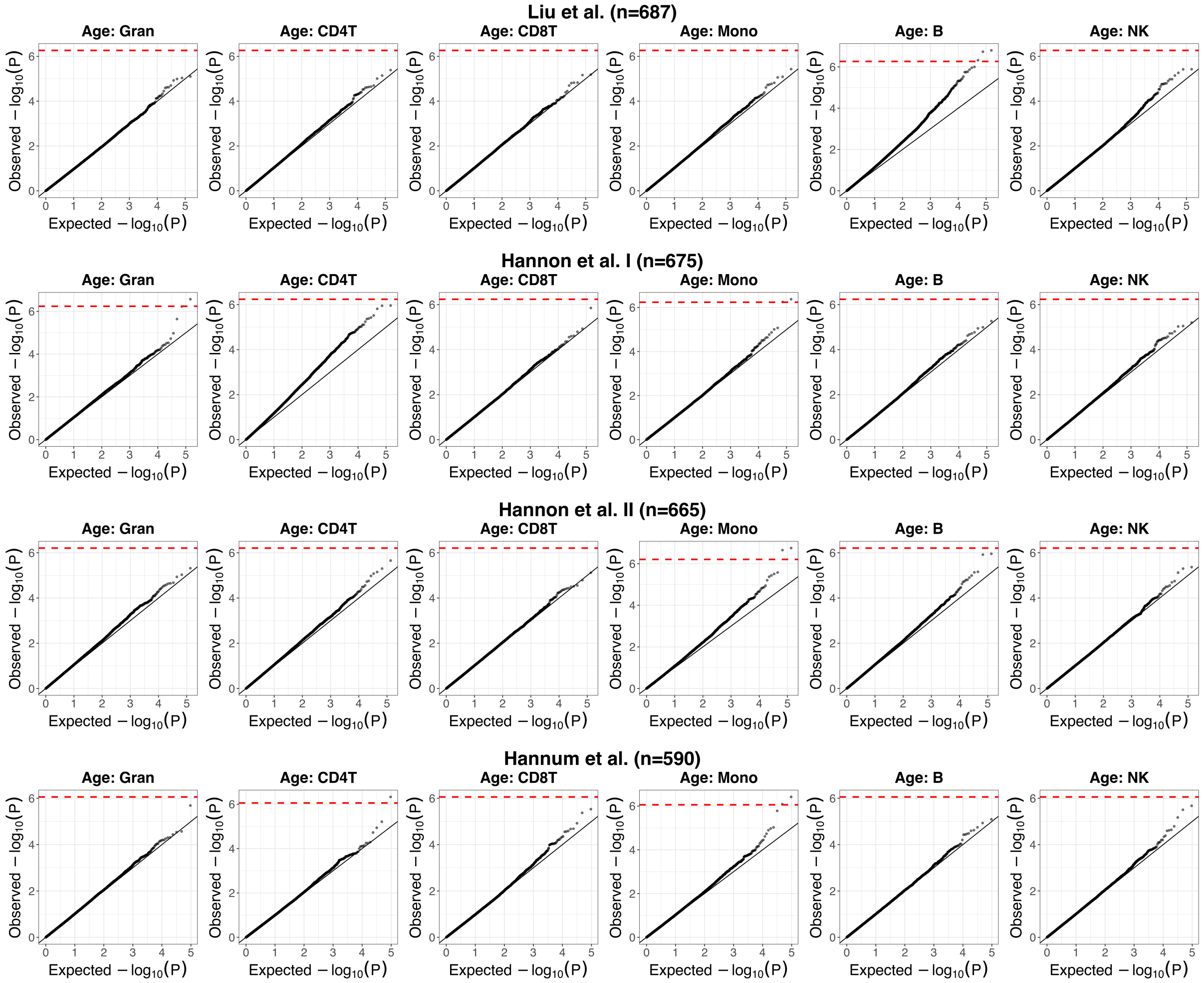

Fig. S27: Evaluation of the null distribution of Unico's asymptotically derived p-values under non-parametric testing for cell-type level differential methylation with age in four whole-blood datasets. Presented are quantile-quantile plots with log-transformed expected p-values versus the observed p-values under permutations of the condition (i.e., under the null). Red horizontal dashed lines indicate the Bonferroni-corrected threshold, adjusting for the number of CpGs and the number of cell types under test.

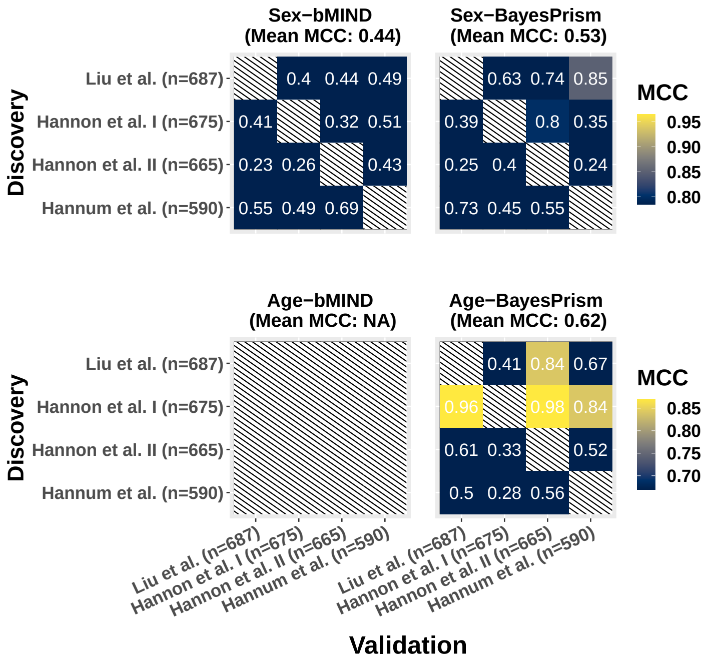

Fig. S28: Calling cell-type level differential methylation with sex and age across four whole-blood DNA methylation datasets using deconvolution methods originally designed for RNA expression. Color gradients represent the Matthews correlation coefficient (MCC) for every possible pairing of two datasets as discovery and validation (Methods). Since bMIND was designed for binary conditions only, it was not evaluated in the age analysis. Both methods leverage the purified cell-type level data from Reinius et al. as prior.

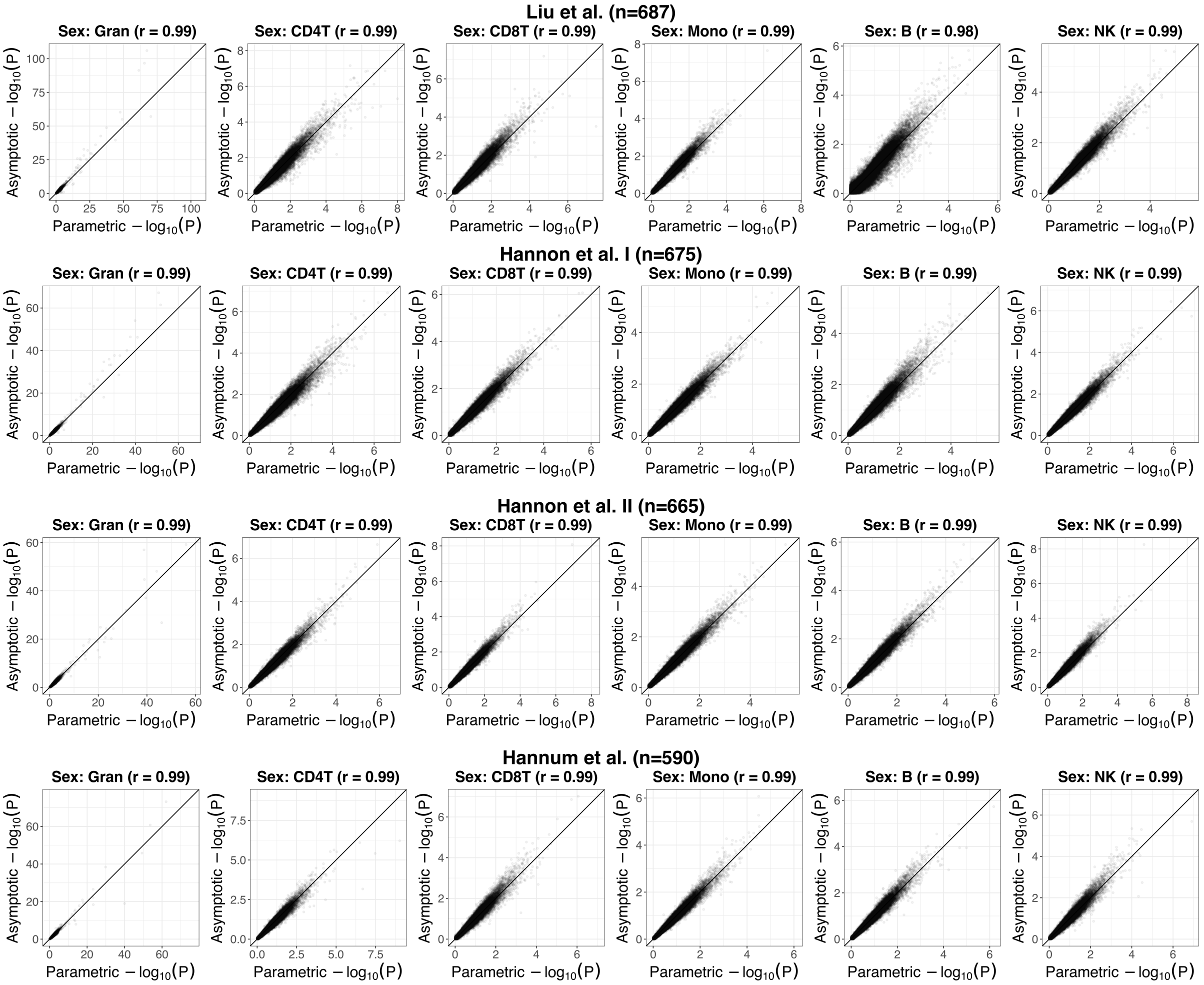

Fig. S29: Evaluation of Unico's asymptotically-derived p-values under non-parametric testing for cell-type level differential methylation with sex in four whole-blood datasets. Presented are scatter plots showing log-transformed p-values under the assumption that methylation levels are normally distributed ("Parametric") versus the corresponding log-transformed p-values of a non-parametric test ("Asymptotic").

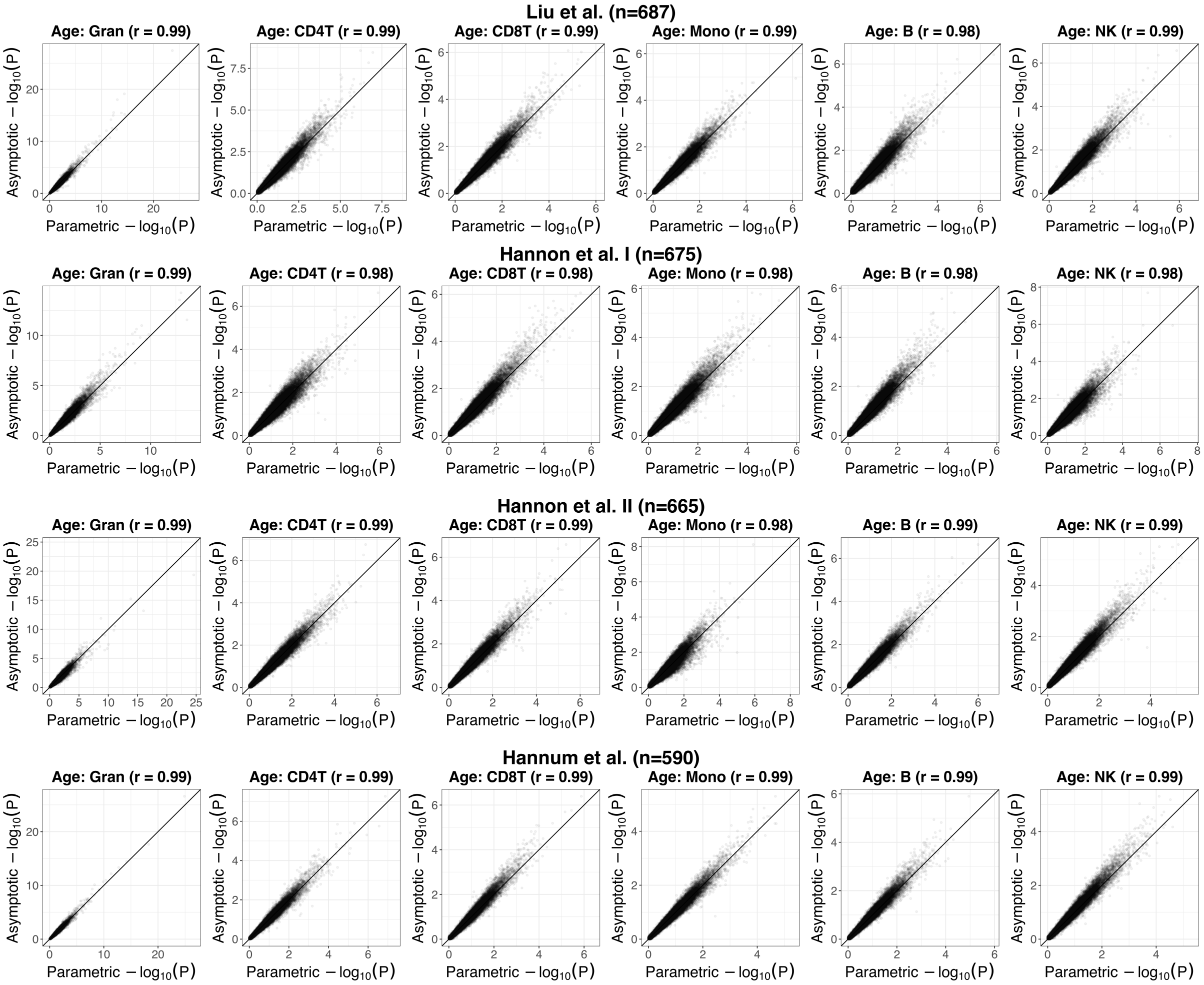

Fig. S30: Evaluation of Unico's asymptotically-derived p-values under non-parametric testing for cell-type level differential methylation with age in four whole-blood datasets. Presented are scatter plots showing log-transformed p-values under the assumption that methylation levels are normally distributed ("Parametric") versus the corresponding log-transformed p-values of a non-parametric test ("Asymptotic").

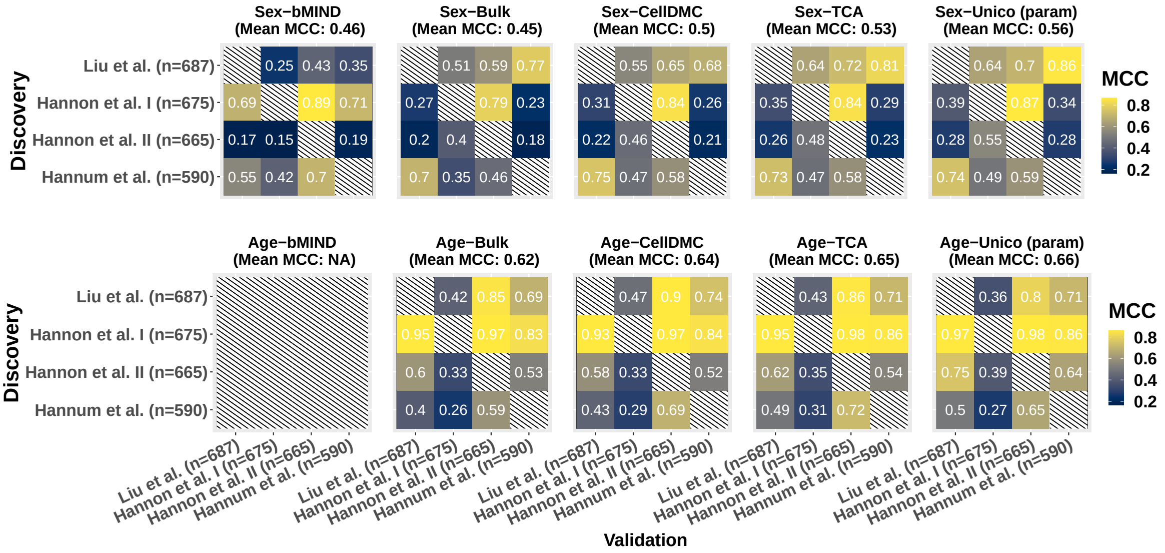

Fig. S31: Consistency in calling tissue-level differential methylation with sex and age across four independent whole-blood DNA methylation datasets. Color gradients represent the Matthews correlation coefficient (MCC) for every possible pairing of two datasets as discovery and validation (Methods). Since bMIND was designed for binary conditions only, it was not evaluated in the age analysis.

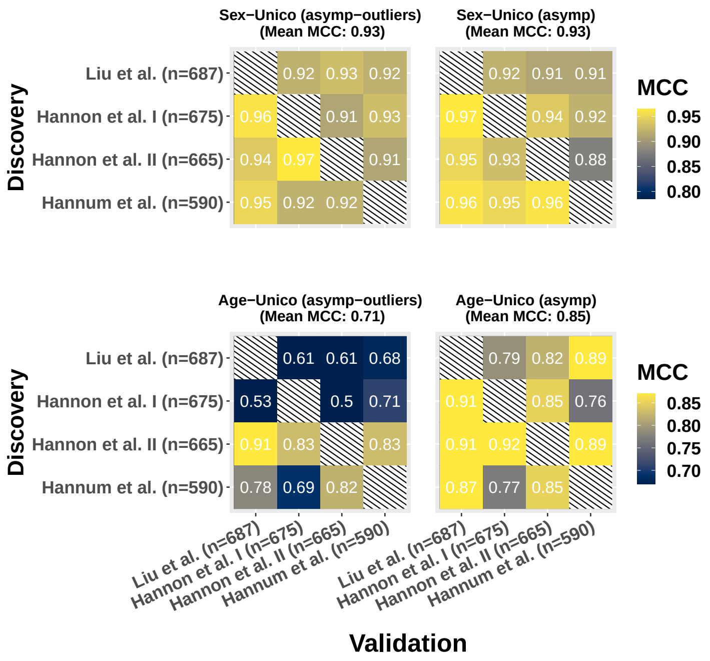

Fig. S32: Effect of outliers on consistency when applying Unico in calling cell-type level differential methylation under asymptotic with sex and age across four independent whole-blood DNA methylation datasets. Color gradients represent the Matthews correlation coefficient (MCC) for every possible pairing of two datasets as discovery and validation (Methods).

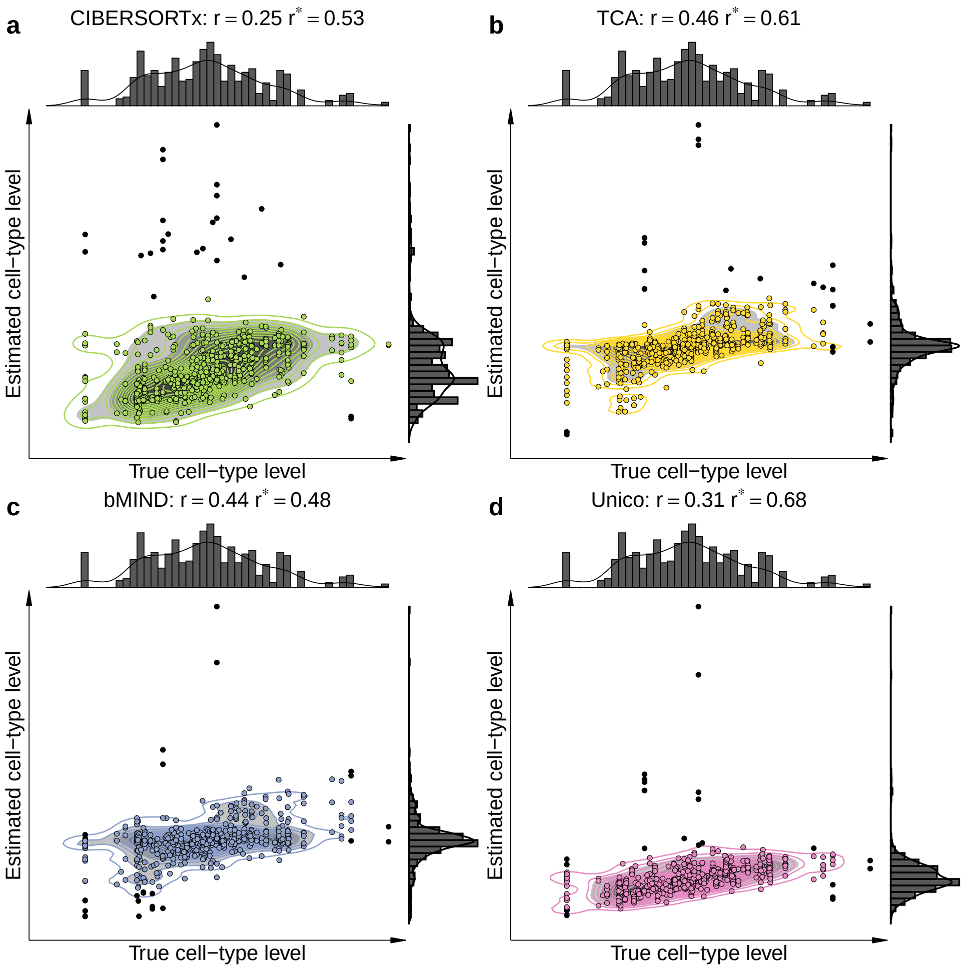

Fig. S33: Example of the potential discrepancy between robust and non-robust correlation across different methods (a-d). Scatter plots show the concordance between the ground truth CD4 expression levels and their deconvolution-derived estimates for the gene *SLC19A1* from the experiment of deconvolving RNA pseudo-bulk mixtures. Black-filled circles indicate 5% of the samples falling outside a 95% confidence ellipsoid (represented by contour lines) and are thus considered outliers. r^* and r indicate the sample linear correlation calculated with and without outliers removed, respectively.

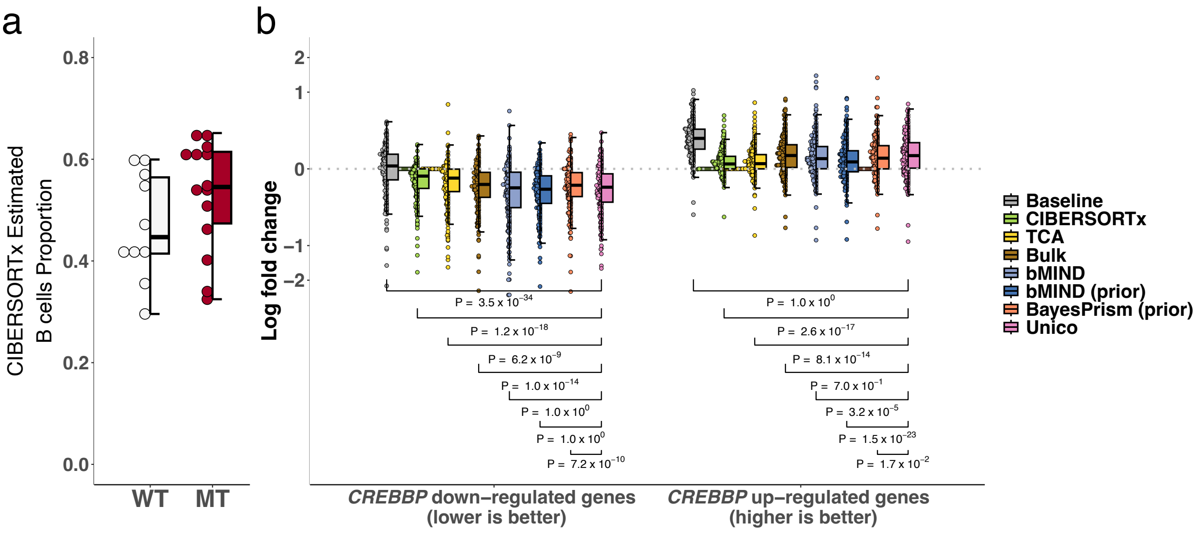

Fig. S34: Deconvolution of bulk FL tumor samples. (a) B cell composition estimates from FL tumor samples with (MT; n=14) and without (WT; n=10) *CREBBP* mutation. (b) Deconvolution of bulk FL tumor samples for evaluating genes that were previously reported as differentially expressed with *CREBBP* mutation in B cells of FL tumors. Presented are the log (basis 2) fold change across 219 down-regulated and 275 up-regulated genes, evaluated on B cell expression estimated by deconvolving the bulk FL samples; pairwise method comparisons against Unico were assessed using a one-sided paired Wilcoxon test. "(prior)" indicates incorporating cell-type level information derived from single-cell data.

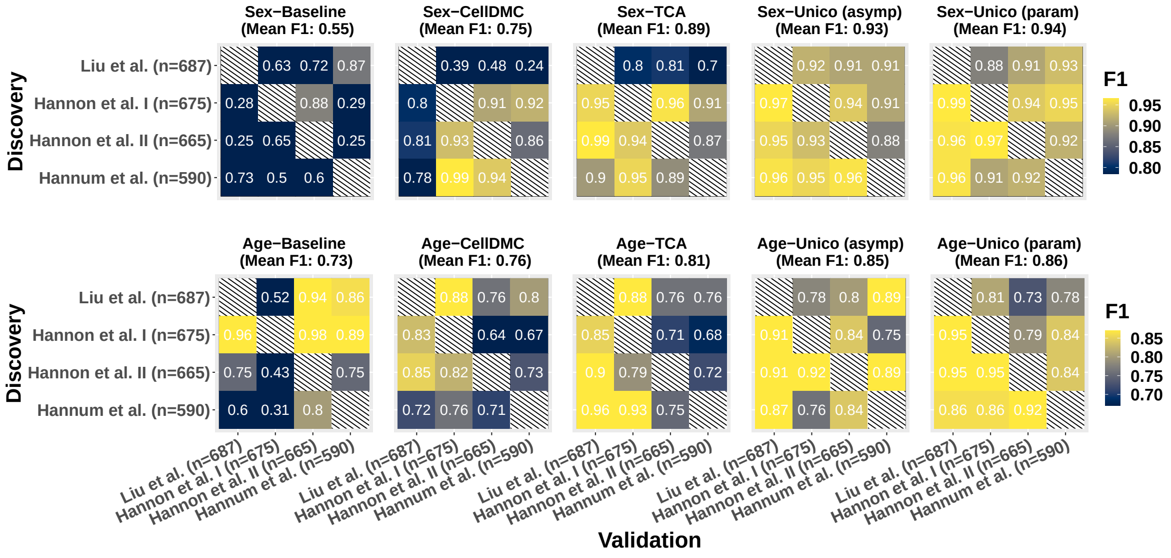

Fig. S35: Consistency in calling cell-type level differential methylation with sex and age across four independent whole-blood DNA methylation datasets. Color gradients represent the F1 score for every possible pairing of two datasets as discovery and validation (Methods).

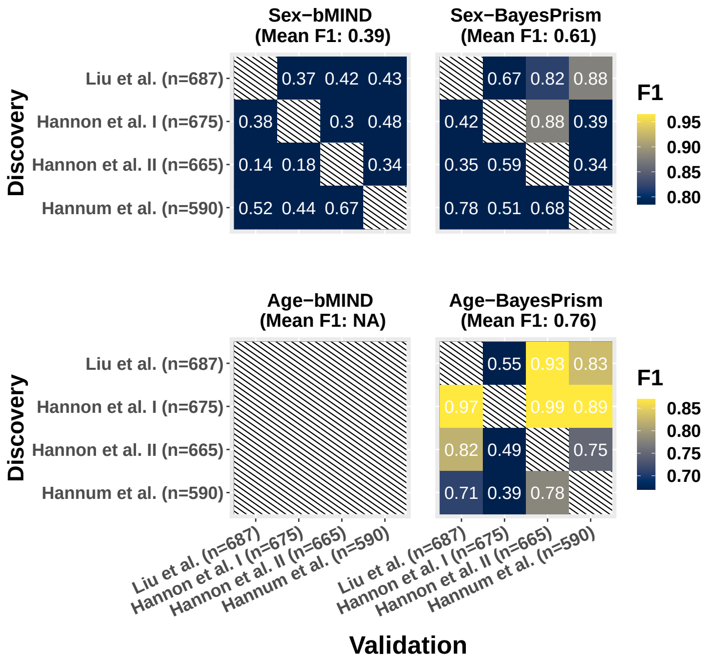

Fig. S36: Calling cell-type level differential methylation with sex and age across four whole-blood DNA methylation datasets using deconvolution methods originally designed for RNA expression. Color gradients represent the F1 score for every possible pairing of two datasets as discovery and validation (Methods). Since bMIND was designed for binary conditions only, it was not evaluated in the age analysis. Both methods leverage the purified cell-type level data from Reinius et al. as prior.

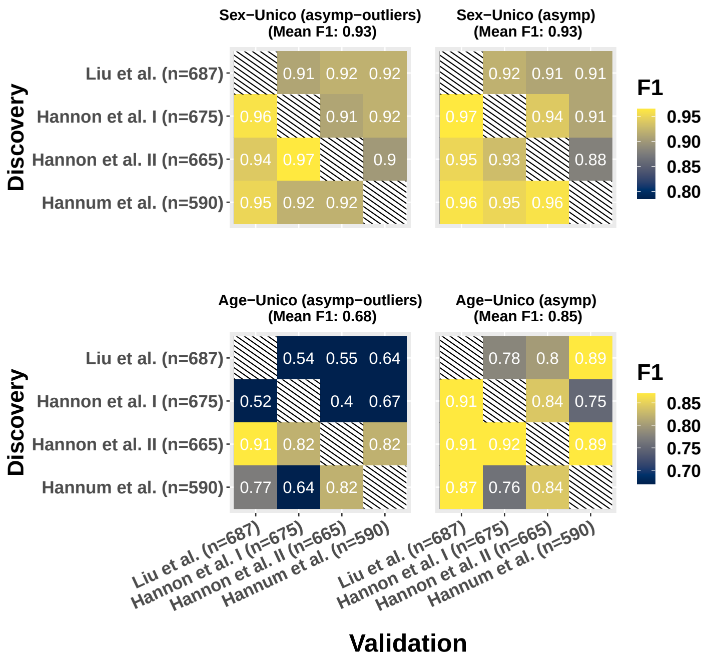

Fig. S37: Effect of outliers on consistency when applying Unico in calling cell-type level differential methylation under asymptotic with sex and age across four independent whole-blood DNA methylation datasets. Color gradients represent the F1 score for every possible pairing of two datasets as discovery and validation (Methods).

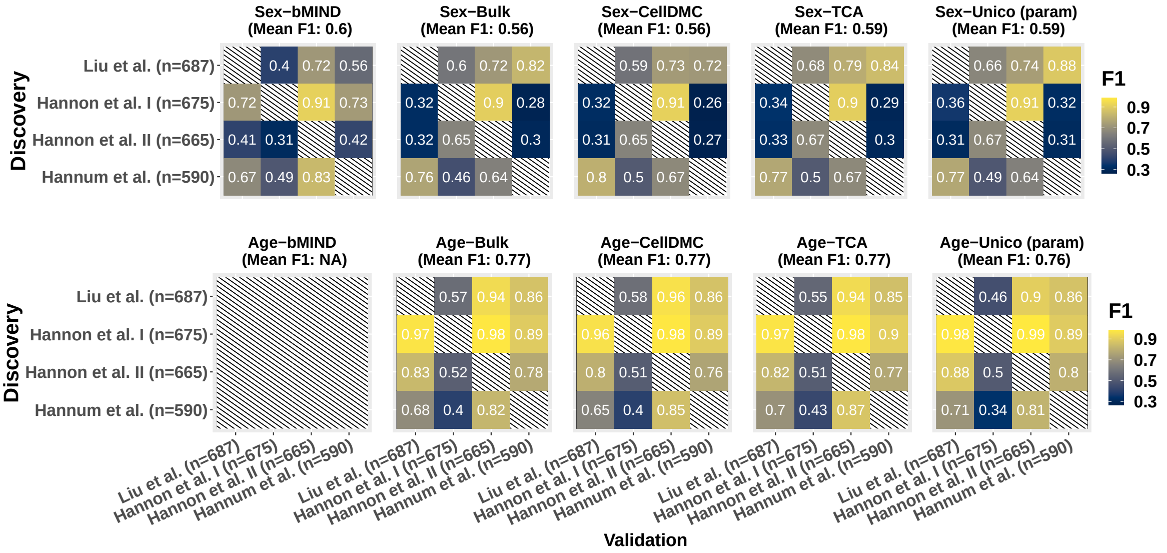

Fig. S38: Consistency in calling tissue-level differential methylation with sex and age across four independent whole-blood DNA methylation datasets. Color gradients represent the F1 score for every possible pairing of two datasets as discovery and validation (Methods). Since bMIND was designed for binary conditions only, it was not evaluated in the age analysis.
