# Supplementary material for: Unico: a unified model for cell-type resolution genomics from heterogeneous omics data
Source: Genome Biol. 2025 Oct 3;26:333. doi: 10.1186/s13059-025-03776-3 (PMC12492706; doi:10.1186/s13059-025-03776-3)
Supplement: Supplementary file 2 — Additional file 2. Supplementary Notes. [file 13059_2025_3776_MOESM2_ESM.pdf]

Unico: a unified model for cell-type resolution genomics from heterogeneous omics data
Supplementary Notes

Zeyuan Johnson Chen[*,1,2], Elior Rahmani [†*,2], Eran Halperin [‡2]

[*]These authors contributed equally

[1]Department of Computer Science, University of California, Los Angeles, CA, USA

[2]Department of Computational Medicine, University of California, Los Angeles, CA, USA

[†]Corresponding Author: eliorrahmani@mednet.ucla.edu

[‡]Corresponding Author: ehalperin@cs.ucla.edu

Contents

S1 Supplementary Methods **3**

S1.1 Unico: uniform cross-omics deconvolution 3

S1.1.1 Unico: distribution-free deconvolution incorporating cell-type covariance Modeling 3

S1.1.2 Unico in the context of previous deconvolution methods 4

S1.2 Proof of Theorem 1: The Unico 3D tensor estimator 6

S1.3 Proof of Theorem 2: Improved capacity to reduce covariance bias 7

S1.4 Optimization of the Unico model 8

S1.5 Generating pseudo-bulk mixtures from scRNA-seq profiles 12

S1.6 Cell-type level differential methylation analysis 13

S1.6.1 Distribution-free statistical testing with Unico 14

S1.6.2 Statistical testing with Unico under a parametric assumption . . . 15

S1.6.3 Statistical testing using competing deconvolution methods 16

S1 Supplementary Methods

S1.1 Unico: uniform cross-omics deconvolution

S1.1.1 Unico: distribution-free deconvolution incorporating cell-type covariance Modeling

Let $X_{ij} \in \mathbb{R}$ be the (tissue-level) bulk level of sample $i \in \{1..., n\}$ in feature $j \in \{1..., m\}$, and let $Z_{ij} = (Z_{ij1}, ..., Z_{ijk})^T \in \mathbb{R}^k$ be a vector of the k cell-type levels of feature j in sample i. The full Unico model assumes:

$$X_{ij} = w_i^T Z_{ij} + (c_i^{(2)})^T \beta_j + e_{ij} \tag{1}$$

$$\text{E}[e_{ij}] = 0, \text{Var}[e_{ij}] = \tau_j^2 \tag{2}$$

$$Z_{ijh} = \mu_{jh} + (c_i^{(1)})^T \gamma_{jh} + \epsilon_{ijh} \tag{3}$$

$$\text{E}[\epsilon_{ijh}] = 0, \text{Var}[\epsilon_{ijh}] = \sigma_{jh}^2 \tag{4}$$

$$(\Sigma_j)_{hq} \equiv \sigma_{jh,jq} \equiv \text{Cov}[Z_{ijh}, Z_{ijq}], \;\; (\Sigma_j)_{hh} \equiv \sigma_{jh,jh} \equiv \sigma_{jh}^2 \tag{5}$$

where $w_i = (w_{i1}, ..., w_{ik})^T \in \mathbb{R}^k$ is a vector of sample-specific cell-type proportions of k cell types that are assumed to compose the studied tissue; $e_{ij} \in \mathbb{R}$ is an i.i.d. component of non-systematic variation with variance $\tau_j^2 \in \mathbb{R}$, reflecting measurement noise; $c_i^{(2)} \in \mathbb{R}^{p_2}$ is a vector of known tissue-level covariate values of sample i that may demonstrate effects on the bulk levels (rather than directly on the underlying cell-type levels), such as batch effects; and $\beta_j \in \mathbb{R}^{p_2}$ is the vector of the corresponding fixed effect sizes. The parameter $\mu_{jh} \in \mathbb{R}$ represents the mean level, specific to feature j and cell type h; $\epsilon_{ijh} \in \mathbb{R}$ is a noise term with mean zero and variance σ_{jh}^2 reflecting the variance of feature j in cell type h; $\Sigma_j \in \mathbb{R}^{k \times k}$ denotes the variance-covariance matrix of $\epsilon_{ij} = (\epsilon_{ij1}, ..., \epsilon_{ijk})^T \in \mathbb{R}^k$ cross cell types, such that $(\Sigma_j)_{hq} \equiv \sigma_{jh,jq}$; $c_i^{(1)} \in \mathbb{R}^{p_1}$ is a vector of known covariate values of sample i that may demonstrate cell-type-specific effects; and $\gamma_{jh} \in \mathbb{R}^{p_1}$ is the vector of the corresponding fixed effect sizes.

Equation (5) reflects our expectation that at least some genomic features will be dif-

ferent yet coordinated between different cell types. Particularly, transcriptional programs are known to persist through multiple differentiation steps, thus leading to differences in expression between cell lineages [1, 2]. Indeed, we observe that a smaller distance on the lineage differentiation tree corresponds to a higher degree of correlation. For example, using large single-cell data from peripheral blood mononuclear cells (PBMCs) (n=118 individuals) [3], we found that the median correlation between monocytes (myeloid lineage) and CD8 T cells (lymphoid lineage) is 0.25 among the top 10,000 most highly expressed genes. In contrast, different lymphoid cell types tend to be more correlated: for example, the median correlation between CD4 T cells and CD8 T cells is 0.71. The median correlation between monocytes and CD4 T cells, B cells, and NK cells is 0.27, 0.30, and 0.37, respectively. The median correlation between CD4 T versus NK cells, CD4 T versus B cells, CD8 T versus NK cells, CD8 T versus B cells, and NK versus B cells is 0.48, 0.40, 0.46, 0.36, and 0.45, respectively.

S1.1.2 Unico in the context of previous deconvolution methods

The first method proposed in the space of deconvolution of transcriptomics is CIBERSORTx [4]. CIBERSORTx uses a heuristic approach based on the key idea that if a data point is used in multiple non-negative matrix factorization problems of different subsets of the data, then its underlying cell-type-specific signals can be approximated by a low-rank combination of the different decomposition products.

BayesPrism, presented in the context of tumor transcriptomics, models the heterogeneity of cell states (or subtypes) within each annotated cell type and leverages single-cell data as prior information [5]. Specifically, it models the counts across genes of a given cell state using a multinomial distribution. It achieves both decomposition and deconvolution by inferring a joint posterior distribution of the cell-state proportions and the underlying 3D tensor via Gibbs sampling. BayesPrism is the only method that models all features in a given sample simultaneously and each sample independently, making it theoretically more suitable for detecting genome-wide shifts in expression/methylation patterns. This characteristic may make it less effective in cell-type level association studies, where the

goal is to identify differential signals of individual features, one at a time, across samples.

TCA is the first deconvolution method proposed for DNA methylation [6]. TCA employs a likelihood-based optimization that was specifically tailored for DNA methylation data, for which the normality assumption is arguably proper for the vast majority of CpGs, if collected from methylation arrays. More concretely, the model follows Equations (1)-(4), while making the additional assumption that the i.i.d. component of variation e_{ij} in Equation (2) is normally distributed and for a given feature j and cell type h the random variables $\{Z_{ijh}\}$ follow a normal distribution with no covariance structure assumed. Unico can, therefore, be viewed as a generalization of the TCA model, as the former does not make distributional assumptions and further models cell-type covariance. Furthermore, since standard decomposition was shown to be a degenerate case of TCA [7], Unico can also be viewed as a generalization of standard decomposition.

Similarly to TCA, the bMIND [8] model follows Equations (1)-(4), and in addition Equation (5), which models cell-type covariance, while making the additional assumption that the components of variation are normally distributed. However, unlike other deconvolution methods, bMIND assumes the data follows a normal distribution after applying a log-transformation (i.e., log-normal). bMIND can incorporate priors for the means and covariances, which can be estimated from either independent single-cell data or the analyzed bulk data. The model is learned using Markov chain Monte Carlo (MCMC) sampling for optimization.

Finally, we would like to note that CODEFACS [9] and MIND [10] were also proposed as deconvolution methods in the context of transcriptomics. CODEFACS is a heuristic method, which was neither open-sourced nor fully detailed by the authors, and MIND is a model-based method that requires multiple measurements from the same samples/individuals, which is beyond the scope of this work. We therefore excluded these two methods from our evaluation and discussion.

S1.2 Proof of Theorem 1: The Unico 3D tensor estimator

Proof. Our goal is estimating $\{z_{ij}\}$, the realizations of $\{Z_{ij}\}$ given data $\{x_{ij}\}$ coming from $\{X_{ij}\}$ using $\mathrm{E}\left[Z_{ij}|\theta_j, X_{ij} = x_{ij}, w_i, c_i^{(1)}, c_i^{(2)}\right]$. We consider a linear transformation $A_{ij} = Z_{ij} + B_{ij}X_{ij}$. Assuming we can construct A_{ij} such that if A_{ij} and X_{ij} are uncorrelated, then they are independent, then given $X_{ij} = x_{ij}$, we can express Z_{ij} using the relation $A_{ij} - B_{ij}X_{ij}$ and calculate the conditional expectation as follows:

$$\begin{aligned}
\mathrm{E}\left[Z_{ij}|\theta_j, x_{ij}, w_i, c_i^{(1)}, c_i^{(2)}\right] &= \mathrm{E}\left[A_{ij} - B_{ij}X_{ij}|\theta_j, x_{ij}, w_i, c_i^{(1)}, c_i^{(2)}\right] &(6)\\
&= \mathrm{E}\left[A_{ij}|\theta_j, x_{ij}, w_i, c_i^{(1)}, c_i^{(2)}\right] - \mathrm{E}\left[B_{ij}X_{ij}|\theta_j, x_{ij}, w_i, c_i^{(1)}, c_i^{(2)}\right]\\
&= \mathrm{E}[A_{ij}|\theta_j, w_i, c_i^{(1)}, c_i^{(2)}] - B_{ij}x_{ij} &(7)\\
&= \mathrm{E}[Z_{ij}|\theta_j, w_i, c_i^{(1)}, c_i^{(2)}] + B_{ij}\left(\mathrm{E}[X_{ij}|\theta_j, w_i, c_i^{(1)}, c_i^{(2)}] - x_{ij}\right)
\end{aligned}$$

X_{ij} is assumed to follow a normal distribution, which means A_{ij}, a linear transformation of X_{ij}, also follows a normal distribution. Since X_{ij}, A_{ij} jointly follow a multivariate normal distribution, Equation (7) can hold if A_{ij} and X_{ij} are uncorrelated, which would imply independence. Thus, we can satisfy Equation (7) by requiring the cross-covariance vector between A_{ij} and X_{ij} to be zero given $\{\theta_j, w_i, c_i^{(1)}, c_i^{(2)}\}$:

$$\mathrm{Cov}[A_{ij}, X_{ij}] = (0, ..., 0) \quad (8)$$

$$\iff \mathrm{Cov}[Z_{ij} + B_{ij}X_{ij}, X_{ij}] = (0, ..., 0) \iff \mathrm{Cov}[Z_{ij}, X_{ij}] + \mathrm{Cov}[B_{ij}X_{ij}, X_{ij}] = (0, ..., 0)$$

$$\iff \mathrm{Cov}[Z_{ij}, X_{ij}] + B_{ij}\mathrm{Var}[X_{ij}] = (0, ..., 0) \iff B_{ij} = -\mathrm{Var}[X_{ij}]^{-1}\mathrm{Cov}[Z_{ij}, X_{ij}] \quad (9)$$

Noting that

$$\mathrm{Cov}[Z_{ijq}, X_{ij}] = \sum_{h=1}^{k} \mathrm{Cov}[Z_{ijq}, w_{ih}Z_{ijh}] = \sum_{h=1}^{k} w_{ih}\sigma_{jq,jh} \quad (10)$$

We thus get

$$B_{ij} = -\mathrm{Var}[X_{ij}]^{-1}\Sigma_j w_i \quad (11)$$

In summary, we get the following Unico estimator:

$$\hat{z}_{ij} = \mathrm{E}[Z_{ij}|\theta_j, w_i, c_i^{(1)}, c_i^{(2)}] + \mathrm{Var}[X_{ij}|\theta_j, w_i, c_i^{(1)}, c_i^{(2)}]^{-1}\Sigma_j w_i(x_{ij} - \mathrm{E}[X_{ij}|\theta_j, w_i, c_i^{(1)}, c_i^{(2)}]) \quad (12)$$

$$\mathrm{E}[X_{ij}|\theta_j, w_i, c_i^{(1)}, c_i^{(2)}] = w_i^T\left(\mu_j + \gamma_j^T(c_i^{(1)})\right) + (c_i^{(2)})^T\beta_j \quad (13)$$

$$\mathrm{Var}[X_{ij}|\theta_j, w_i, c_i^{(1)}, c_i^{(2)}] = \mathrm{Sum}\left((w_i w_i^T) \odot \Sigma_j\right) + \tau_j^2 \quad (14)$$

where $\gamma_j = (\gamma_{j1}, ..., \gamma_{jk}) \in \mathbb{R}^{p_1 \times k}$ is a matrix composed of the vectors $\{\gamma_{jh}\}$, the $\odot$ operator is the Hadamard product of two matrices, and the Sum$(\cdot)$ operator is a summation across all entries of a matrix. □

S1.3 Proof of Theorem 2: Improved capacity to reduce covariance bias

Proof. Consider a simple scenario where for some gene j, $\mu_j = 0, \Sigma_j = I_k$, $\tau_j^2 = 0$, and we have no covariates. In this case, the TCA estimator for cell type h becomes [6]:

$$\hat{z}_{ijh}^{\text{TCA}} = \frac{w_{ih} x_{ij}}{\|w_i\|_2^2} \tag{15}$$

While TCA does not explicitly model cell-type covariance, we can use this estimator to calculate the covariance between the TCA-estimated values of two cell types $h \neq l$ (denote $\hat{\sigma}_{jh,jl}$) without observing an actual data point x_{ij}. For a given sample i with w_i we get

$$\text{Cov}\left[\hat{z}_{ijh}^{\text{TCA}} | X_{ij}, \hat{z}_{ijl}^{\text{TCA}} | X_{ij}\right] = \frac{w_{ih} w_{il} \text{Var}[X_{ij}]}{\|w_i\|_2^4} = \frac{w_{ih} w_{il}}{\|w_i\|_2^2} \tag{16}$$

Now, while we treat the cell-type proportions $\{w_i\}$ as fixed for the samples in the data, they have some distribution in the population. Since cell-type proportions are bounded to the range $[0, 1]$, the mean of that distribution exists. Hence, combining the information across samples, we get

$$\hat{\sigma}_{jh,jl}^{\text{TCA}} = \frac{1}{n} \sum_{i=1}^{n} \frac{w_{ih} w_{il}}{\|w_i\|_2^2} \xrightarrow{p} c_{hl} \tag{17}$$

where c_{hl} is some constant, specific to cell types h, l yet independent of j and thus does not necessarily reflect the true correlation between the two cell types. In contrast, similarly considering the Unico estimator from Equations (12)-(14) under the same assumptions, we get:

$$\hat{\sigma}_{jh,jl}^{\text{Unico}} = \frac{1}{n} \sum_{i=1}^{n} \frac{((\Sigma_j)_h^T w_i)((\Sigma_j)_l^T w_i)}{\text{Var}[X_{ij}]} \xrightarrow{p} f(\Sigma_j) \tag{18}$$

where $(\Sigma_j)_h$ is the h-th column of Σ_j and $f(\Sigma_j)$ is some function of the variance matrix of gene j. It is easy to see that if $\Sigma_j = I_k$ then $f(\Sigma_j) = c_{hl}$ and Unico reduces to TCA.

However, for genes with a non-trivial covariance matrix, the Unico estimator, in principle, has more capacity to alleviate the limitation of TCA in reflecting true covariances between cell types. □

S1.4 Optimization of the Unico model

Optimization setup. We estimate the unknown parameters of the model by following concepts from the Generalized Method of Moments (GMM) [11]. The GMM framework allows us to learn the parameters of a model by solving equations (moment conditions) that match population moments (or, more generally, a function of population moments) with their corresponding data-derived sample moments. More concretely, let $f(\Theta, X_n) = f_1(\Theta, X_n), ..., f_d(\Theta, X_n)$ be d moment conditions defined over parameters Θ, where $X_n = (x_1, ..., x_n)$ are observations coming from the assumed model and $d > |\Theta|$. If the moment conditions satisfy $\forall 1 \leq l \leq d$: $\mathrm{E}[f_l(\Theta, X_n)] = 0$ then an asymptotically consistent estimator of Θ is given by [11]:

$$\hat{\Theta} = \underset{\Theta}{\operatorname{argmin}}\, f(\Theta, X_n)^T \hat{U}_n f(\Theta, X_n) \tag{19}$$

where $\hat{U}_n$ is a positive definite (PD) weighting matrix. In the Unico model we treat X_{ij} as a random variable with mean and variance that depend on gene-specific parameters (θ_j), as well as on sample-specific cell-type proportions w_i. As a result, the variables $\{X_{ij}\}$, in general, have different distributions; however, these distributions are all parameterized by the same set θ_j of constant size. This leads us to construct a separate moment condition for each sample, which results in a multi-equation GMM problem with shared parameters across the Equation [12]. Since each moment condition is represented by a single sample the optimization of the Unico model eventually reduces to solving multiple weighted least squares problems.

Below, we provide a detailed derivation of Unico's optimization procedure. A summary of the optimization procedure is provided in Algorithm 1.

Learning the means of the model. We start by learning the means, which can be estimated independently from the variances and covariances. Specifically, let

$$\phi_j = (\mu_{j1}, ..., \mu_{jk}, \gamma_{j11}, ..., \gamma_{jkp_1}, \beta_{j1}, ..., \beta_{jp_2})$$

be a vector of the means of gene j, for each sample i in the data we define $f_i(\phi_j, x_{ij}) = x_{ij} - \mu_{X_{ij}}$, such that $\mu_{X_{ij}} \equiv \mathrm{E}[X_{ij}]$; clearly, $\mathrm{E}[f_i(\phi_j, x_{ij})] = 0$.

In theory, under a typical GMM estimation, setting the weights matrix $\hat{U}_n$ in Equation (19) to be the inverse of the empirical variance-covariance matrix of the moment conditions results in an asymptotically efficient estimator [11]. In our case, given that different moment conditions are based on independent individuals, this corresponds to setting $\hat{U}_n \in \mathbb{R}^{n \times n}$ as a diagonal matrix with values $\{\hat{u}_{ij} = (x_{ij} - \hat{\mu}_{X_{ij}})^{-2}\}_i$ on the diagonal; thus, reducing Equation (19) to the following weighted least squares problem for estimating ϕ_j:

$$\hat{\phi}_j = \underset{\phi_j}{\mathrm{argmin}} \sum_{i=1}^{n} \hat{u}_{ij} \left(x_{ij} - \phi_j^T s_i\right)^2 \tag{20}$$

$$s_i = (w_{i1}, ..., w_{ik},\ w_{i1}c_{i1}^{(1)}, ..., w_{i1}c_{ip_1}^{(1)}, ..., w_{ik}c_{i1}^{(1)}, ..., w_{ik}c_{ip_1}^{(1)},\ c_{i1}^{(2)}, ..., c_{ip_2}^{(2)}) \tag{21}$$

Indeed, we know from large sample theory of weighted least squares problems that this is an asymptotically consistent and efficient estimator. However, in practice, we choose to forfeit asymptotic efficiency for practical considerations in finite data. Specifically, we wish to avoid very high values of $\hat{u}_{ij}$, which can greatly affect the solution by relying on just a small number of data points with extreme weights. In practice, we address this by modifying the definition of $\hat{u}_{ij}$ to allow only down-weighting of samples:

$$\hat{u}_{ij} = \min\left(1, (x_{ij} - \hat{\mu}_{X_{ij}})^{-2}\right) \tag{22}$$

Notably, Equation (22) relies on an estimate of $\mu_{X_{ij}}$, which requires estimating the parameters ϕ_j. However, these are exactly the parameters that we wish to estimate in Equation (20). We resolve this by employing an alternating optimization: we first solve Equation (20) while setting $\forall i : \hat{u}_{ij} = 1$, and then, given initial estimates of ϕ_j, we follow Equation (22) for constructing the weights for the subsequent optimization of Equation (20). This procedure can be followed by more iterations until convergence, much like

in a standard feasible generalized least squares. In practice, we further add constraints requiring $\{\mu_{jh}\}$ to be non-negative.

Solving Equation (20) requires us to learn a total of $k(1+p_1)+p_2$ parameters for each gene. This is a constant number of parameters compared with the number of data points used for learning these parameters (n). The optimization of each gene is independent, which makes the problem feasible in principle. However, in practice, any application of the above optimization should be mindful of the balance between the sample size and this number of parameters.

Learning the variances and covariances of the model. Once the algorithm converges to a set of means ($\hat{\phi}_j$), we move on to the estimation of the variances and covariances of gene j. Let $\psi_j = \text{diag}(\Sigma_j)$ and let $\xi_j = \text{tri}(\Sigma_j)$ be a vector of the elements in the lower diagonal entries of the matrix Σ_j (excluding the diagonal elements). We follow the same approach we take for estimating the means, which yields the following optimization problem:

$$\hat{\psi}_j, \hat{\xi}_j, \hat{\tau}_j^2 = \underset{\psi_j, \tau_j^2}{\text{argmin}} \sum_{i=1}^{n} \hat{v}_{ij} \left((x_{ij} - \hat{\mu}_{X_{ij}})^2 - \psi_j^T s_i' - 2\xi_j^T s_i'' - \tau_j^2 \right)^2 \tag{23}$$

$$s_i' = w_i \odot w_i, s_i'' = \text{tri}(w_i w_i^T) \tag{24}$$

$$\hat{v}_{ij} = \min\left(1, \left((x_{ij} - \hat{\mu}_{X_{ij}})^2 - \hat{\sigma}_{X_{ij}}^2\right)^{-2}\right), \quad \sigma_{X_{ij}}^2 \equiv \text{Var}[X_{ij}] \tag{25}$$

In order to direct the inference towards a good solution we further wish to constrain $\hat{\psi}_j, \hat{\xi}_j$ to form an estimate of Σ_j that is positive definite (PD). We, therefore, reformulate the above optimization problem as follows. Consider the Cholesky decomposition $\Sigma_j = L_j L_j^T$ where L_j is a lower triangular matrix with positive values on its diagonal. Instead of directly estimating the elements of Σ_j by solving Equation (23)-(25), we guarantee that $\hat{\Sigma}_j$ is PD by estimating the non-zero elements of L_j. Specifically, we express Σ_j in the optimization problem through L_j, which results in the following optimization problem:

$$\hat{L}_j, \hat{\tau_j}^2 = \underset{L_j, \tau_j^2}{\text{argmin}} \sum_{i=1}^{n} \hat{v}_{ij} \left((x_{ij} - \hat{\mu}_{X_{ij}})^2 - d_j^T s_i' - 2l_j^T s_i'' - \tau_j^2 \right)^2 \tag{26}$$

$$\text{s.t.}, \forall h \in \{1, ..., k\} : L_{hh} > 0 \tag{27}$$

where

$$d_j = \mathrm{diag}(L_j L_j^T), l_j = \mathrm{tri}(L_j L_j^T) \tag{28}$$

$$s_i' = w_i \odot w_i, s_i'' = \mathrm{tri}(w_i w_i^T) \tag{29}$$

$$\hat{v}_{ij} = \min\left(1, \left((x_{ij} - \hat{\mu}_{X_{ij}})^2 - \hat{\sigma}^2_{X_{ij}}\right)^{-2}\right), \quad \sigma^2_{X_{ij}} \equiv \mathrm{Var}[X_{ij}] \tag{30}$$

Finally, given $\hat{L}_j$, we set $\hat{\Sigma}_j = \hat{L}_j \hat{L}_j^T$. The above problem is non-convex, however, it requires learning small independent problems of $\binom{k}{2} + k + 1$ parameters for each gene (where k is a small constant; e.g., 5 cell types). Similarly to the estimation of the means, we apply an alternating optimization procedure to overcome the need for an estimate $\hat{\sigma}^2_{X_{ij}}$ in Equation (30).

Algorithm 1 Optimization procedure for **Unico**.

for each feature j present in genomic data X **do**
  $\forall i \quad \hat{u}_{ij} \leftarrow 1$ ▷ Initialize weights
  while `not converged` **do** ▷ Estimate the means
    $\hat{\phi}_j \leftarrow \mathrm{argmin}_{\phi_j} \sum_{i=1}^n \hat{u}_{ij} \left(x_{ij} - \phi_j^T s_i\right)^2$
    for each sample i **do**
      $\hat{u}_{ij} \leftarrow \min\left(1, (x_{ij} - \hat{\mu}_{X_{ij}})^{-2}\right)$
    end for
  end while
  $\forall i \quad \hat{v}_{ij} \leftarrow 1$ ▷ Initialize weights
  while `not converged` **do** ▷ Estimate variances and covariances
    $\hat{L}_j, \hat{\tau}_j{}^2 \leftarrow \mathrm{argmin}_{L_j, \tau_j^2} \sum_{i=1}^n \hat{v}_{ij} \left((x_{ij} - \hat{\mu}_{X_{ij}})^2 - d_j^T s_i' - 2 l_j^T s_i'' - \tau_j^2\right)^2$
    $\hat{\Sigma}_j \leftarrow \hat{L}_j \hat{L}_j^T$
    for each sample i **do**
      $\hat{v}_{ij} \leftarrow \min\left(1, \left((x_{ij} - \hat{\mu}_{X_{ij}})^2 - \hat{\sigma}^2_{X_{ij}}\right)^{-2}\right)$
    end for
  end while
end for

S1.5 Generating pseudo-bulk mixtures from scRNA-seq profiles

We simulated pseudo-bulk PBMC expression profiles using single-cell PBMCs from the Stephenson et al. study [3] and pseudo-bulk lung expression profiles using data from the Human Lung Cell Atlas (HLCA) [13]. In order to account for sequencing depth, gene counts were converted to counts-per-million (CPM) in every cell before averaging all cells of type h from sample i to generate a cell-type-specific pseudo-bulk expression pattern.

We estimated per-sample cell-type proportions for both single-cell datasets by calculating cell counts per individual sample for all modeled cell types ($k \in \{5, 7\}$ for PBMC and $k \in \{4, 6\}$ for HCLA) and normalizing them to sum up to 1 per sample. Average proportions were 31.8%, 19.5%, 17.7%, 16.7%, 14.2% for the top five main cell types in PBMC: CD4 T cells, NK cells, CD8 T cells, monocytes and B cells, respectively (k=5 scenario). Monocytes can be further stratified to 14.7% CD14 monocytes and 2.0% CD16 monocytes, and B cells can be further stratified to 13.4% canonical B cells and 0.8% plasma cells (k=7 scenario). In the lung data, we only considered samples collected from parenchyma tissues, which demonstrated average proportions of 62.5%, 23.8%, 8.4%, and 5.4% for the top four main cell types: immune cells, epithelial cells, endothelial cells, and stromal cells, respectively (k=4 scenario). Immune cells can be further stratified to 43.5% myeloids and 17.4% lymphoid cells, and epithelial cells can be further stratified to 18.8% alveolar epithelium and 5.6% airway epithelium cells (k=6 scenario).

We generated every pseudo-bulk sample by mixing cell-type proportions from one sample (randomly drawn with replacement) and all cell-type level profiles of a single sample (randomly drawn with replacement). In addition, we drew a few noisy versions of the cell-type proportion estimates from the following Dirichlet distribution: $\mathrm{Dir}(\alpha w_i)$, where w_i denotes the ground truth proportion of the sample and $\alpha \in \{100, 50, 25, 10, 5, 2.5\}$ controls the noise level. Large α forces the distribution to be concentrated around the true proportion while smaller ones allow more derivation. To avoid sampling extremely lowly expressed genes, we restricted our sampling space to the top 10,000 most expressed genes, evaluated based on the gene-specific sum of average expression across cell types. In cases where genes demonstrated too low expression in a certain cell type (mean or

variance less than 10^{-4} after excluding outliers that are 2 standard deviations away from the mean), we added a small non-negative Gaussian noise $\mathcal{HN}(0, 10^{-4})$. This was done to improve numerical stability and to ensure the gene-level correlation matrix remains of full rank when calculating cell-type covariance entropy. Per simulated dataset, we carried out the above-mentioned sampling strategy independently for 600 genes, and for varying numbers of sample sizes (50, 100, 250, 500, or 1000 samples). Per experiment, we repeated the mixture sampling procedure 20 times.

For the purpose of evaluation, both the pseudo-bulk mixtures and cell-type specific expression profiles were scaled by the standard deviation of each gene, which we calculated from the pseudo-bulk data so that the variance across different genes is roughly comparable. Omitting this standardization step would induce spuriously high correlation scores for algorithms that simply estimate the relative scale of the parameters correctly.

Finally, for every cell type, we excluded genes with associated cell-type expression profiles that demonstrated (after scaling) mean or variance ≤ 0.1 from correlation calculation and multiple linear regression analysis with pseudo-bulk profile. This exclusion alleviated the risk of evaluating deconvolution methods against merely artificially added noise as described earlier or extremely noisy counts. Of note, in general, CIBERSORTx estimates cell-type proportions from the bulk input. Here, however, we directly provided it with the ground truth cell-type proportion of the mixtures, as provided to all other methods we benchmarked.

S1.6 Cell-type level differential methylation analysis

The Unico model naturally allows statistical testing for cell-type level associations by incorporating a phenotype of interest as a cell-type level covariate $\{c_i^{(1)}\}$ and testing whether its effect is non-zero (i.e., whether $\gamma_{jh} \neq 0$ for a given feature j and cell-type h). This does not require us to explicitly estimate the underlying 3D tensor of cell-type levels. Instead, we can estimate effects directly following the model in Equations (1)-(5). As we describe below, we can take either a non-parametric approach or a parametric

approach for such statistical testing.

S1.6.1 Distribution-free statistical testing with Unico

Given the estimated model parameters (means, variances, and covariances), we can derive asymptotic p-values for cell-type-specific effect sizes. Let S be a design matrix formed by stacking $s_1, s_2, ..., s_n$ following Equation (21) as the rows, and let $Q_j = \text{diag}(q_{1j}^2, ..., q_{nj}^2)$ be a diagonal weighting matrix, where q_{ij} follows Equation (40), we solve:

$$\hat{\phi}_j^{\text{asym}} = \underset{\phi_j}{\text{argmin}}(x_j - S\phi_j)^T Q_j (x_j - S\phi_j) \tag{31}$$

where $x_j = (x_{1j}, ..., x_{nj})$. We get a weighted least squares (WLS) problem, which is characterized by the following analytical solution and asymptotic distribution of the estimator $\hat{\phi}_j^{\text{asym}}$:

$$\hat{\phi}_j^{\text{asym}} = (S^T Q_j S)^{-1} S^T Q_j x_j \tag{32}$$

$$\hat{\phi}_j^{\text{asym}} \xrightarrow{d} N(\phi_j, (S^T Q_j S)^{-1} S^T Q_j \text{Var}[f(\phi_j, X_{ij})] Q_j S (S^T Q_j S)^{-1}) \tag{33}$$

where $f(\phi_j, X_{ij}) = x_{ij} - \mu_{X_{ij}}$ and its empirical variance is $\text{Var}[f(\phi_j, X_{ij})] = (x_{ij} - \mu_{X_{ij}})^2$. This can be written more compactly as:

$$\hat{\phi}_j^{\text{asym}} \xrightarrow{d} N(\phi_j, (S^T Q_j S)^{-1} S^T Q_j^* S (S^T Q_j S)^{-1}) \tag{34}$$

$$(Q_j^*)_{ii} = (Q_j)_{ii}^2 (x_{ij} - \mu_{X_{ij}})^2 \tag{35}$$

where $(Q_j)_{ii}$ denotes the i-th element on the diagonal of Q_j. Now, following Slutsky's Theorem, we get:

$$\frac{(\hat{\phi}_j^{\text{asym}})_p}{\text{SE}(\hat{\phi}_j^{\text{asym}})_{pp}} \xrightarrow{d} N(0, 1) \tag{36}$$

$$\text{SE}(\hat{\phi}_j^{\text{asym}})_{pp} = \sqrt{[(S^T Q_j S)^{-1} S^T Q_j^* S (S^T Q_j S)^{-1})]_{pp}} \tag{37}$$

where $(\hat{\phi}_j^{\text{asym}})_p$ denotes the p-th entry of $\hat{\phi}_j^{\text{asym}}$. Given Equation (36), statistical testing becomes straightforward using the cumulative distribution function of the standard normal distribution.

The standard error $\mathrm{SE}(\hat{\phi}_j^{\text{asym}})$ is a function of the moment conditions $f(\hat{\phi}_j, X_{ij})$, each calculated based on a single data point, making it highly sensitive to outliers. To prevent outlier data points from dominating the solution, we excluded 5% of the samples with the lowest empirical variance $\mathrm{Var}[f(\hat{\phi}_j)]$.

Empirically, we observe that outlier values in the cell-type level covariates $c^{(1)}$ composing the design matrix S can greatly affect the estimated effect sizes $(\hat{\phi}_j^{\text{asym}})_p$ and standard errors $\mathrm{SE}(\hat{\phi}_j^{\text{asym}})_{pp}$. To address this, we excluded samples with extreme deviation from the mean, measured based on $c^{(1)}$. Concretely, we calculated the Mahalanobis distance (MD) of the continuous covariates in $c_i^{(1)}$ of each sample i from the mean of the covariates in the data. We removed samples with $MD \geq \chi^2_{0.95,df}$, where df corresponds to the number of continuous covariates. This amounts to removing samples with covariate values outside a theoretical 95% quantile (assuming the covariates are normally distributed). In our cell-type level DM testing with age, this step removed samples falling in the tails of the age group with respect to each dataset: those over 62 in the Liu et al. data, below 17 or above 73 in the Hannum et al. data, over 62 in the Hannon et al. I data, and below 20 or over 69 in the Hannon et al. II data.

S1.6.2 Statistical testing with Unico under a parametric assumption

To adapt Unico for cell-type DM parametric testing under the assumption that the data follows a normal distribution, we assume:

$$Z_{ij} \sim \mathcal{N}\left(\mu_j + \gamma_j^T(c_i^{(1)}), \Sigma_j\right) \tag{38}$$

$$X_{ij} \sim \mathcal{N}\left(w_i^T(\mu_j + \gamma_j^T(c_i^{(1)})) + (c_i^{(2)})^T\beta_j, \mathrm{Sum}\left((w_i w_i^T) \odot \Sigma_j\right) + \tau_j^2\right) \tag{39}$$

For a given CpG j under test, Equation (39) corresponds to a heteroskedastic regression problem with $\{x_{ij}\}_i$ as the dependent variable and $\{w_i\}, \{w_i c_i^{(1)}\}, \{c_i^{(2)}\}$ as the independent variables. This view allows us to perform statistical testing by solving a generalized least squares problem using a standard linear regression framework. Concretely, we scale every methylation sample i by the inverse of its estimated standard deviation:

$$q_{ij} := \left(\mathrm{Sum}\left((w_i w_i^T) \odot \hat{\Sigma}_j\right) + \hat{\tau}_j^2\right)^{-0.5} \tag{40}$$

where $\hat{\Sigma}_j, \hat{\tau}_j^2$ correspond to the cell-type covariance and residual variance parameters estimated under the non-parametric model optimization of Unico. More specifically, we scale both the dependent and independent variables of sample i by q_{ij} and fit a standard linear regression model. Statistical testing then becomes straightforward: marginal cell-type-level effects can be evaluated using a standard t statistic, and tissue-level effects can be evaluated using a partial F statistic that quantifies the joint effect across all cell types.

S1.6.3 Statistical testing using competing deconvolution methods

Testing for DM with sex and age should consider the effect of these demographics on methylation levels and not vice versa [7]. Assuming this model directionality is natural within the Unico model, which can evaluate the effect of sex and age as cell-type level covariates in (3). Similarly, we applied TCA, CellDMC, and our baseline model to evaluate the effects of sex and age on cell-type level methylation. BayesPrism does not offer any integrated association testing framework. We hence adopted an approach similar to the one we used for the baseline model (Methods), where we directly performed statistical testing on the 3D tensor, estimated using purified cell-type level data from Reinius et al. [14] as prior.

bMIND, on the other hand, required a different approach. The bMIND implementation includes two modes for association testing (both of which only support binary phenotypes; i.e., only sex in our case). The first mode models the appropriate direction of effect, as indicated above. However, it relies on MCMC sampling, which renders it infeasible for epigenome-wide association studies; particularly, in our case, execution time is expected to be over 24 hours for a single CpG, assuming 30 threads, in order to pass a Bonferroni-corrected threshold. We thus opted for the alternative mode of bMIND, which the bMIND authors recommended and is computationally feasible. Specifically, we first estimated the 3D tensor with prior derived from the purified cell-type level data from Reinius et al. [14] and performed testing directly on the tensor values. We estimated cell-type-level effects and corresponding p-values based on logistic regression models, using the tensor's cell-type-level profiles and covariates as the independent variables and

sex as the dependent variable.

In addition to evaluating cell-type-level DM, we further tested for tissue-level DM. Similarly to Unico, TCA allows calculating such tissue-level p-values [6]. In contrast, CellDMC only evaluates and reports summary statistics for marginal cell-type level tests [15]. We, therefore, implemented a tissue-level test for CellDMC by learning a restricted linear regression model without the cell-type interaction terms and performing a partial F-test based on the original unrestricted model. For bMIND, p-values were derived for the tissue-level tests from a multivariate analysis of covariance (MANCOVA) with sex as the independent variable and cell-type level profiles as dependent variables with $\{c_i^{(1)}\}$ and $\{c_i^{(2)}\}$ covariates adjusted.

BayesPrism was excluded in this evaluation as there is no straightforward way to implement tissue-level testing with the appropriate directionality from only the tensor estimates. Finally, when evaluating tissue-level DM based on a straightforward analysis of the bulk data, we performed a standard linear regression analysis directly on the bulk mixture with bulk data as the dependent variable and cell-type proportions, as well as all covariates, as independent variables. P-values for tissue-level effects were derived using a standard t statistic.

References

1. Kluger Y, Tuck DP, Chang JT, Nakayama Y, Poddar R, Kohya N, et al. Lineage specificity of gene expression patterns. Proc. Natl. Acad. Sci. U.S.A. 2004;101(17):6508–6513.
2. Novershtern N, Subramanian A, Lawton LN, Mak RH, Haining WN, McConkey ME, et al. Densely interconnected transcriptional circuits control cell states in human hematopoiesis. Cell. 2011;144(2):296–309.

3. Stephenson E, Reynolds G, Botting RA, Calero-Nieto FJ, Morgan MD, Tuong ZK, et al. Single-cell multi-omics analysis of the immune response in COVID-19. Nat. Med. 2021;27(5):904–916.
4. Newman AM, Steen CB, Liu CL, Gentles AJ, Chaudhuri AA, Scherer F, et al. Determining cell type abundance and expression from bulk tissues with digital cytometry. Nat. Biotechnol. 2019;37(7):773–782.
5. Chu T, Wang Z, Pe'er D, and Danko CG. Cell type and gene expression deconvolution with BayesPrism enables Bayesian integrative analysis across bulk and single-cell RNA sequencing in oncology. Nat. Cancer. 2022;3:505–517.
6. Rahmani E, Schweiger R, Rhead B, Criswell LA, Barcellos LF, Eskin E, et al. Cell-type-specific resolution epigenetics without the need for cell sorting or single-cell biology. Nat. Commun. 2019;10(1):1–11.
7. Rahmani E, Jew B, and Halperin E. The Effect of Model Directionality on Cell-Type-Specific Differential DNA Methylation Analysis. Front. Bioinform. 2022;1:792605.
8. Wang J, Roeder K, and Devlin B. Bayesian estimation of cell type–specific gene expression with prior derived from single-cell data. Genome Res. 2021;31(10):1807–1818.
9. Wang K, Patkar S, Lee JS, Gertz EM, Robinson W, Schischlik F, et al. Deconvolving clinically relevant cellular immune cross-talk from bulk gene expression using CODEFACS and LIRICS stratifies patients with melanoma to anti–PD-1 therapy. Cancer Discov. 2022;12(4):1088–1105.
10. Wang J, Devlin B, and Roeder K. Using multiple measurements of tissue to estimate subject-and cell-type-specific gene expression. Bioinformatics. 2020;36(3):782–788.
11. Hansen LP. Large sample properties of generalized method of moments estimators. Econometrica. 1982;50(4):1029–1054.
12. Hayashi F. Econometrics. Princeton: Princeton University Press; 2000
13. Sikkema L, Ramírez-Suástegui C, Strobl DC, Gillett TE, Zappia L, Madissoon E, et al. An integrated cell atlas of the lung in health and disease. Nat. Med. 2023;29:1563–1577.

14. Reinius LE, Acevedo N, Joerink M, Pershagen G, Dahlén SE, Greco D, et al. Differential DNA methylation in purified human blood cells: implications for cell lineage and studies on disease susceptibility. PLoS One. 2012;7(7):e41361.

15. Zheng SC, Breeze CE, Beck S, and Teschendorff AE. Identification of differentially methylated cell types in epigenome-wide association studies. Nat. Methods. 2018;15(12):1059–1066.
